# Revision of the genus *Lichtwardtia* Enderlein in Southeast Asia, a tale of highly diverse male terminalia (Diptera, Dolichopodidae)

**DOI:** 10.3897/zookeys.798.28107

**Published:** 2018-11-21

**Authors:** Chufei Tang, Ding Yang, Patrick Grootaert

**Affiliations:** 1 Department of Entomology, College of Plant Protection, China Agricultural University, Beijing 100094, China China Agricultural University Beijing China; 2 Entomology, Royal Belgian Institute of Natural Sciences, Vautierstraat 29, B-1000 Brussels, Belgium Royal Belgian Institute of Natural Sciences Brussels Belgium; 3 National Biodiversity Centre, NParks and Lee Kong Chian Natural History Museum, National University of Singapore, Singapore National University of Singapore Singapore Singapore

**Keywords:** *
Lichtwardtia
*, new species, Oriental, revision

## Abstract

In the present paper the Oriental species of the genus *Lichtwardtia* Enderlein, 1912 are revised based on the type material of known species and new material from Singapore and Cambodia. A re-description and illustration of the holotype female of *Lichtwardtiaziczac* (Wiedemann, 1824) is given but since it has been described on the basis of a female only and its provenance India Orientalis is only a vague indication of its type locality, it is considered as a nomen dubium. All the species put as junior synonyms by Becker (1922) of *L.ziczac* are re-established to their original status with diagnosis: *Lichtwardtiapolychroma* (Loew, 1864) and *Lichtwardtiaformosana* Enderlein, 1912. However, *L.coxalis* is now also considered as a nomen dubium since the original description is too short to distinguish it from other species and the holotype female is lost. In addition a re-description and illustrations of *L.hirsutiseta* (de Meijere, 1916) are provided. Eight new species for science are described and illustrated: *Lichtwardtiacambodiensis* Tang & Grootaert, **sp. n.** (Cambodia), *Lichtwardtiaconspicabilis* Tang & Grootaert, **sp. n.** (Cambodia), *Lichtwardtiainfuscata* Tang & Grootaert, **sp. n.** (Cambodia), *Lichtwardtiamonstruosa* Tang & Grootaert, **sp. n.** (Cambodia), *Lichtwardtianodulata* Grootaert & Tang, **sp. n.** (Singapore), *Lichtwardtiasemakau* Grootaert & Tang, **sp. n.** (Singapore) and *Lichtwardtiasingaporensis* Grootaert & Tang, **sp. n.** (Singapore). *Lichtwardtiazhangae* Tang & Grootaert, **sp. n.** (Bali, Indonesia) is a new name for the species described by Zhang, Masunaga & Yang, 2009, as *Lichtwardtiaziczac* (Wiedemann, 1824). There are only a few good diagnostic non-genitalic characters for the species, but the male terminalia are distinctive, from simple to very complicated and armed structures. A key is given to the species of the Oriental region. Barcodes are provided for the Singaporean species.

Although *Lichtwardtia* is a common genus in Southeast Asia it is generally not abundant locally. It is often found in anthropogenic disturbed habitats only. Four species are recorded from Singapore while eight species are sympatric and very abundant at the locality of Siem Reap in Cambodia.

## Introduction

With its bayonet-like vein M and long soft hairs on the apical segment of the arista-like stylus, the genus *Lichtwardtia* Enderlein is easily recognised as such, but assigning specimens to the correct species is another story. Even the validity of the genus *Lichtwardtia* itself has been uncertain since its establishment (Becker 1922, Brooks 2005, Grichanov and Brooks 2017). Brooks (2005) recognised *Lichtwardtia* as a subgroup of *Dolichopus* with very delicate morphological analyses. However, we have some concerns about the study: Only *Lichtwardtiaangularis* (Macquart, 1842) and a species not confirmed to species level was used for building the character matrix. Meanwhile, *Lichtwardtiaangularis* was a species assigned by a female holotype, which might have brought some potential risk. Besides, only three *Dolichopus* species were used while there were four *Hercostomus*, four *Paraclius*, five *Gymnopternus* and six *Tachytrechus* included. The sampling size has obvious bias, which might have caused the paraphyletic status of *Dolichopus*. Last but not least, a unique specialisation of male terminalia is firstly reported, with various denticles on the hypandrium and the phallus. This character was not shown in the matrix while it is rather unusual. Though *Lichtwardtia* was suggested to be paraphyletic with *Dolichopus* in Brooks 2005, its monophyly was confirmed as a group based on three homoplasious character states, including the possession of an S-shaped bend in vein M, the T-shaped ejaculatory apodeme, and the feather-like stylus. Notably, these features are quite rare in not only Dolichopodinae, but also Dolichopodidae. Apparently the other species of *Dolichopus*, if we consider *Lichtwardtia* a group of *Dolichopus*, do not possess these features, which could help distinguishing the other genera. Therefore we restore the genus *Lichtwardtia*, as was by done by Selivanova et al. 2010 and Grichanov and Brooks 2017. Beyond morphological data, some molecular studies that could prove the monophyly of *Lichtwardtia* are in preparation for publication.

There are 22 species known in the world: 16 from the Afrotropical Region (Grichanov 1998, 2004), four species are present in the Oriental Region (Yang et al. 2011; Zhang et al. 2009), and two species are known from Australia (Yang et al. 2006; Bickel 2008; Grichanov 2011).

The taxonomy of *Lichtwardtia* suffered from the description of species based on females only. Pairing males to these females became almost impossible as females of *Lichtwardtia* barely differ without careful observation and dissection, which also raises the concern of synonyms. Typically, many species were set to be the synonym of the iconic species *L.ziczac* (Wiedemann) that itself was described on the basis of a female. Its provenance “India orientalis” is vague since in the past this area extended from Pakistan in the West to New Guinea in the East. In addition, the single type of *L.coxalis* Kertész, 1901 is probably destroyed (Foldvary and Papp 2007). Zhang et al. (2009) were the first to illustrate detailed male terminalia of Oriental *Lichtwardtia*. They assumed that a male according to the re-description by Becker (1922) belonged to the iconic *L.ziczac* or *L.zickzack* as Becker (1922) spelled it. The problematic previous studies caused the subsequent prudence in describing new species. Male characters, which are used to define the species here, are very distinctive, especially the terminalia. In addition, COI barcoding and NGS barcoding (Meier et al. 2016) are used to support morpho-species concepts and to associate females with males in difficult cases, at least for the Singaporean species.

Techniques to study ancient DNA might one day elucidate its status and that of related species. Wing interference patterns (WIPs) might be a tool which would help pairing the specimens. It has been proved useful on the taxonomy of *Campsicnemus*, which is another genus of Dolichopodidae. However, this could not fully support the identification independently. Often the pattern arrangements between females and males are still at ‘similar’ level, not exactly the same. Here we do not consider this a reliable method for identification because there is no standard at this stage. Meanwhile, the WIPs of *Lichtwardtia* between different species seem not as distinguishably different as those of *Campsicnemus*. The level of similarity that could confirm the pairing to the level of species should not only be determined by the study of one single genus but extended to other genera.

In the present paper we revise the known Oriental fauna and add eight species new for science. With illustrations of the male terminalia we hope to provide a framework to recognise well-defined morpho-species. In addition, some (COI) barcodes (600 bp) and NGS barcodes (313 bp) are provided for all the species with fresh specimens available (uploaded to GenBank, with accession number MH536852–MH536856). In contrast to what we originally thought, the genus *Lichtwardtia* is as diverse in the Oriental region as it is in the Afrotropical region (Grichanov and Brooks 2017). A high diversity and complexity of the morphology of the phallus and hypandrium of the genus are noticed for the first time, from very simple structures, over spiny, saw-toothed to heavily armed intromitted (phallus) organs as well as guiding organs (hypandrium) bearing hooks and thorns.

## Materials and methods

**Figure 1.**
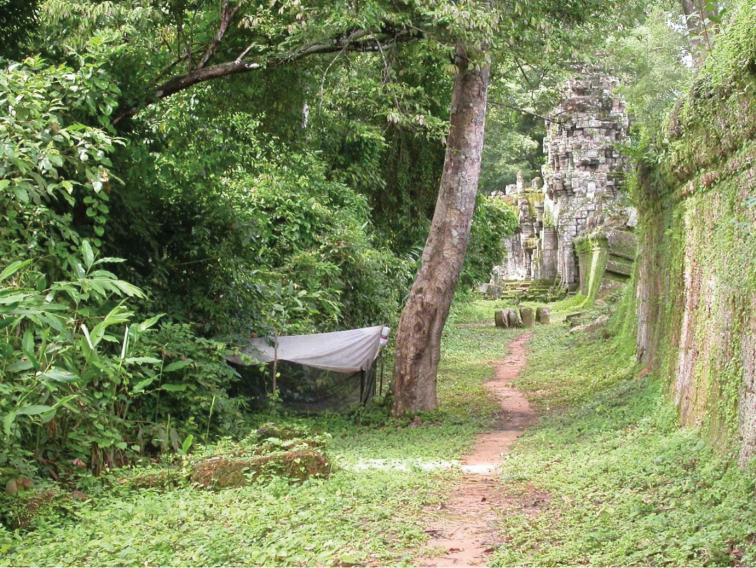
Malaise trap at the site of the Preah Khan Temple (Angkor, Siem Reap, Cambodia).

In addition to the type material of the previously described species, new *Lichtwardtia* specimens were collected during a two-year survey with Malaise traps at the temple site of Preah Khan, a temple at the Angkor site (Siem Reap, Cambodia) and in the garden of the Sam Vesna Centre in Siem Reap. The site of Preah Khan was situated along a path bordering a secondary forest (Figure 1), with elevation ca. 40–45 m. The material from Singapore and Thailand were collected with Malaise traps and by sweep netting as part of a survey of the mangroves and forests of Singapore (Grootaert 2018). The sites from Singapore are all with elevation ca. 0–5 m. All collected specimens were preserved and described in 75% ethanol unless specifically noted.

Morphological terminology for adult structures mainly follows McAlpine (1981), and the structures of the male genitalia follow Cumming and Wood (2009). Photos were taken with a Canon EOS 600D camera at the Royal Belgian Institute of Natural Sciences and then stacked by Helicon Focus 6.0 or with the Visionary Digital BK Plus Lab System at the Laboratory for Evolutionary Biology (**NUS**).The type material is deposited at the Royal Belgian Institute of Natural Sciences (**RBINS**, Brussels) and at the Lee Kong Chian Natural History Museum (**LKCNHM**) in Singapore.

COI barcoding was done following the techniques described in Lim et al. (2010) and by Meier et al. (2016). The evolutionary history was inferred by using the Maximum Likelihood method based on the Hasegawa-Kishino-Yano model (Hasegawa et al. 1985). The bootstrap consensus tree inferred from 1000 replicates (Felsenstein, 1985) is taken to represent the evolutionary history of the taxa analysed (Felsenstein, 1985). Branches corresponding to partitions reproduced in less than 50% bootstrap replicates are collapsed. The percentage of replicate trees in which the associated taxa clustered together in the bootstrap test (1000 replicates) is shown next to the branches (Felsenstein, 1985). Initial tree(s) for the heuristic search were obtained automatically by applying the Maximum Parsimony method. A discrete Gamma distribution was used to model evolutionary rate differences among sites (six categories (+G, parameter = 0.2535)). The rate variation model allowed for some sites to be evolutionarily invariable ([+I], 5.69% sites). The analysis involved nine nucleotide sequences. There were a total of 649 positions in the final dataset. Evolutionary analyses were conducted in MEGA7 (Kuma et al. 2016). Additional barcodes were downloaded from GenBank with the accession number in front of the species name on the phylogeny, are mostly from Sikes et al. (2017).

As for the other old type material seen in this study, we preferred not to dissect the specimens for examination as the features on the body are enough for identification and the specimens might be examined in the near future with less destructive techniques. Abbreviations in the text are as follows:

**a** anterior;

**acr** acrostichal bristle(s);

**ad** anterodorsal bristle(s);

**av** anteroventral bristle(s);

**C** costal vein;

**d** dorsal bristle(s);

**dc** dorsocentral bristle(s);

**hm** postpronotal bristle(s);

**npl** notopleural bristle(s);

**p** posterior bristle(s);

**pa** postalar bristle(s);

**pd** posterodorsal bristle(s);

**pm** presutural supraalar bristle(s);

**pv** posteroventral bristle(s);

**sa** postsutural supraalar bristle(s);

**sc** scutellar bristle(s);

**sr** presutural intraalar bristle(s);

**v** ventral bristle(s).

Abbreviations on the figures are as follows:

**ap** apical bristle(s);

**bvl** basoventral epandrial lobe;

**dsur** dorsal surstylus;

**eb** epandrial bristles;

**hy** hypandrium;

**L** left hand side;

**ph** phallus;

**pg** postgonite;

**R** right hand side;

**vsur** ventral surstylus;

**sur** surstylus.

## Taxonomy

### Family DOLICHOPODIDAE Latreille, 1809

#### Subfamily Dolichopodinae Latreille, 1809

##### Genus Lichtwardtia

Taxon classificationAnimaliaDipteraDolichopodidae

Enderlein, 1912

Lichtwardtia Enderlein, 1912: 406. Type species: Lichtwardtiaformosana Enderlein, 1912 (original designation).
Vaalimyia Curran, 1926: 398. Type species: Vaalimyiaviolacea Curran, 1926 [= Dolichopusangularis Macquart, 1842] (original designation).

###### Notes.

The generic synonymy list is as given by Yang et al. (2005) except that *Lichtwardtiaformosana* Enderlein, 1912 was considered to be a junior synonym of *L.ziczac* (Wiedemann 1824); it is confirmed a valid species in this work.

###### Diagnosis.

Small to medium-sized species (3.5–5.0 mm). Head: overall dark metallic green including vertex, with thick pale pollinosity; face wide, slightly narrow at middle, slightly raised from lateral view, frons and face both with thick pale pollinosity that hiding the ground colour, face parallel sided, but slightly narrowed near mid-length. Hairs and bristles on head black but lower postocular bristles pale. Vertex flat. Ocellar tubercle distinct. With pairs of strong vertical and diverging ocellar bristles present, with weak postvertical bristles which are approx. a half-length of vertical bristles. Antenna wholly or mostly yellow; scape with short dorsal bristles, longer than pedicel; arista-like stylus dorsal, two-segmented, with feather-like long hairs on apical segment. Eyes dichoptic, with hairs between facets. Clypeus long and wide.

Thorax: dark metallic blue-black. Metapleuron with one narrow black stripe. Hairs and bristles on thorax black. Acr biseriate, hair-like. Five strong pairs of dc. With one pa, two sa, one sr, one hm, one pm, two npl. Propleuron with long curved bristle just above base of fore coxa. With hairs anterior to spiracle. Scutellum with two pairs sc, apical pair long and strong, basal pair short and weak. Mid and hind coxae with outer bristle(s). Fore femur without distinct bristle. Mid and hind femora each with preapical bristle. Hind femur thick, 5.0 times longer than wide. Hind tarsomere I with strong dorsal bristles, shorter than hind tarsomere II.

Wing: usually clear, sometimes with faint brown clouding around distal vein M and dm-cu crossvein, occasionally partly smoky. Vein costa sometimes widened at the joint with R_1_, with various callus. M with fading M_2_, M_1_ with one short subvein. Crossvein dm-cu straight. Vein M joining margin just before apex. CuAx around 1.0.

Abdomen: metallic green (in Oriental), nearly 1.5 times longer than wide, with pale pollinosity. Hairs and bristles on abdomen black.

Male terminalia: Epandrium distinctly longer than wides. Epandrial lobe with three long pale bristles Cercus nearly triangular, margin rounded, usually pale with broad black outer margin, with weak digitations around outer margin, with black simple or specialised marginal bristles on digitations. Hypandrium and phallus various, often with various denticles.

##### Lichtwardtia nodulata

Taxon classificationAnimaliaDipteraDolichopodidae

group

###### Diagnosis.

The cercus is more or less triangular, bordered by some strong marginal bristles that are strongly flattened or truncate. All cercus have one or two large inside bristles near the dorsal border. The postgonite is broad and the tip is bifid. The tip of the phallus is ventrally denticulate and the hypandrium is a simple tube.

##### Lichtwardtia cambodiensis

Taxon classificationAnimaliaDipteraDolichopodidae

Tang & Grootaert sp. n.

http://zoobank.org/B81C8619-7CE6-4E98-B533-FE53867568EC

Figs 2 , 3

###### Material.

**Holotype male** (coll. RBINS): CAMBODIA: Siem Reap prov., Angkhor, Preah Khan Temple, 24 January–21 February 2006, Malaise trap in secondary forest (leg. Oul Yothin). **Paratypes** (all coll. RBINS): CAMBODIA: Same provenance as holotype, 13 males, 2 females, 24 January–21 February 2006; 3 males, 4–11 April 2006; 6 males, 8 February–7 March 2006; 8 males, 28 November–7 December 2005; 9 males, 8 March–5 April 2005; 46 males, 17–24 February 2005; 1 male, 1 female, Siem Reap prov., Bakheng, 23–31 October 2005, Malaise trap in secondary forest (leg. Oul Yothin).

###### Diagnosis.

Wing entirely hyaline; with a slight swelling of the costa where R_1_ merges with costa. Postpedicel mainly dark yellow but blackish on apically half, nearly as long as wide. Mid coxa with a dark brown stripe anteriorly, a paler brown band posteriorly. Hind coxa entirely yellow. Cercus rounded, marginal bristles black, not flattened nor on tubercles. Hypandrium simple and smooth, with no denticle; phallus with double rows of spinules on ventral half.

###### Description.

**Male.***Body* length 3.4–3.7 mm, wing 3.4–3.5 × 1.0 mm.

*Head* dark metallic green, with thick pale pollinosity; face slightly raised, frons and face both with thick pale pollinosity, gradually narrowed downward. Hairs and bristles on head black but lower postocular bristles pale. Antenna dark yellow; postpedicel blackish on apical half, blunt apically, nearly as long as wide arista-like stylus dark brown, nearly as long as width of head, feather-like, with long pubescence, basal portion 0.2 times as long as apical portion. Proboscis dark yellow, with black hairs; palpus dark yellow, with1 short black apical bristle.

*Thorax* dark green, with pale grey pollinosity. Hairs and bristles on thorax black; five strong dc, ten pairs of long acr. Scutellum with two pairs sc, apical pair long and strong, basal pair short and weak. Legs mainly yellow; but mid coxa with two large brown square spots laterally: a dark brown stripe anteriorly, a paler brown band posteriorly; mid and hind tarsomeres I–II blackish on tip, mid and hind tarsomere III blackish at apical half, mid and hind tarsomeres IV–V wholly black. Fore and mid coxae anteriorly with rows of bristle-like hairs, fore coxa anteriorly with three long strong marginal bristles, mid coxa with two outer bristles and two marginal bristles, hind coxa with two outer bristles, basal one strong, apical one smaller. Mid trochanter with three short ap, all dorsal. Hind trochanter with two weak ap, one dorsal, one posteroventer. Fore femur with one short weak av. Mid femur with one strong pd and one preapical pv in normal strength. Hind femur with one strong pd at apical quarter. Fore tibia with four ad (of which one strong and preapical), one av and three ap. Mid tibia with two ad, three pd, one pv, and four ap. Hind tibia with four ad, four pd, two pv and three ap. Fore tarsomere I with one short av at base. Hind tarsomere I with one strong ad and two short av. Relative lengths of tibia and five tarsomeres of legs LI : 7.0 : 5.0 : 2.5 : 2.0 : 1.2 : 1.0; LII : 15.0 : 7.5 : 3.8 : 3.0 : 2.0 : 1.5; LIII : 16.2 : 7.0 : 7.5 : 5.0 : 2.2 : 1.5. Wing nearly hyaline, veins brown. Costa slightly widened at the joint with R_1_. M with fading M_2_, M_1_ with one short subvein. Crossvein dm-cu straight. CuAx ratio 1.0. Lower calypter pale with black hairs. Haltere pale.

*Abdomen* metallic green, with pale pollinosity. Hairs and bristles on abdomen black.

*Male terminalia* (Figure 3): Epandrium 1.6 times longer than wide; epandrial lobe with three long pale bristles. Ventral surstylus and dorsal surstylus both with five short nail-like ap (Figure 3A). Cercus nearly triangular, margin rounded, pale with broad black outer margin, with weak digitations around outer margin, with black simple marginal bristles on digitations, with two relatively strong bristles on lateral digitations at apical half. Hypandrium simple and smooth, with no denticle. Phallus with double rows of spinules on ventral half.

**Female.** Similar to male in size and morphology except for the male terminalia.

###### Etymology.

This new species is named after the country (Cambodia) where it was found.

###### Comments.

*Lichtwardtiacambodiensis* sp. n. belongs to the *L.nodulata* group and thus is related to *L.dentalis* and *L.semakau* sp. n. The latter two species both have distinctly darkened and flattened marginal bristles on the cercus while *L.cambodiensis* sp. n. only has thin, and weak marginal bristles on the cercus. Further, *L.cambodiensis* has a weak swelling on the joint point of wing vein R_1_ and costa, whereas the swelling is distinct in *L.dentalis*, and absent in *L.semakau*.

###### Distribution.

Cambodia.

**Figure 2.**
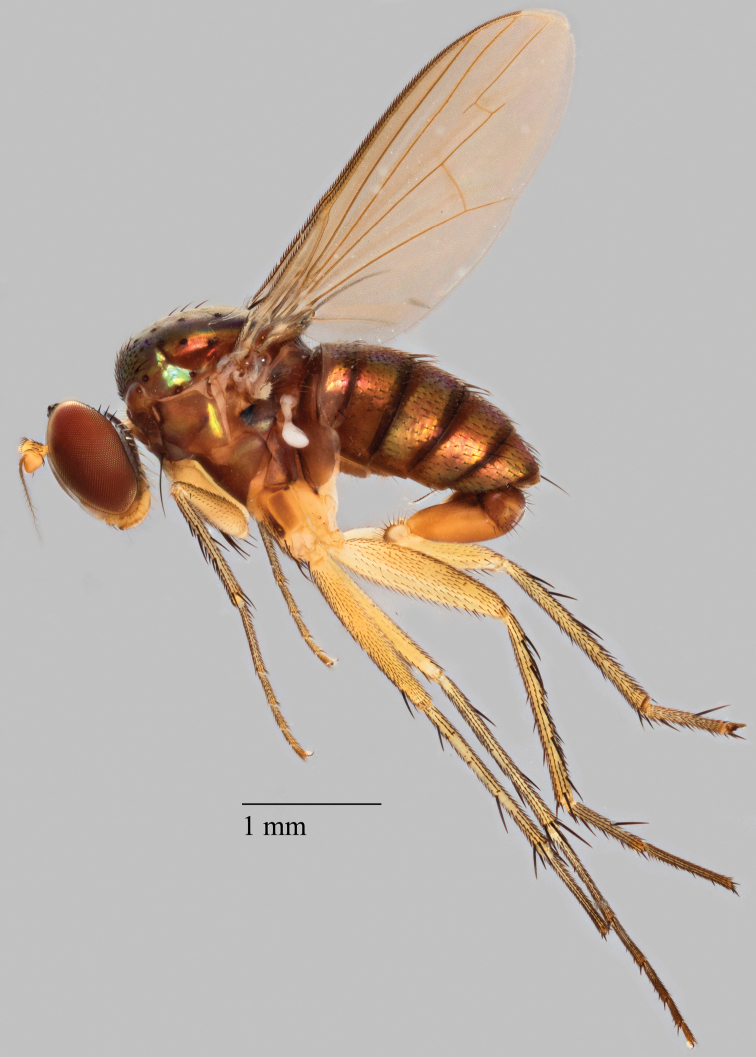
*Lichtwardtiacambodiensis* Tang & Grootaert, sp. n. habitus male.

**Figure 3.**
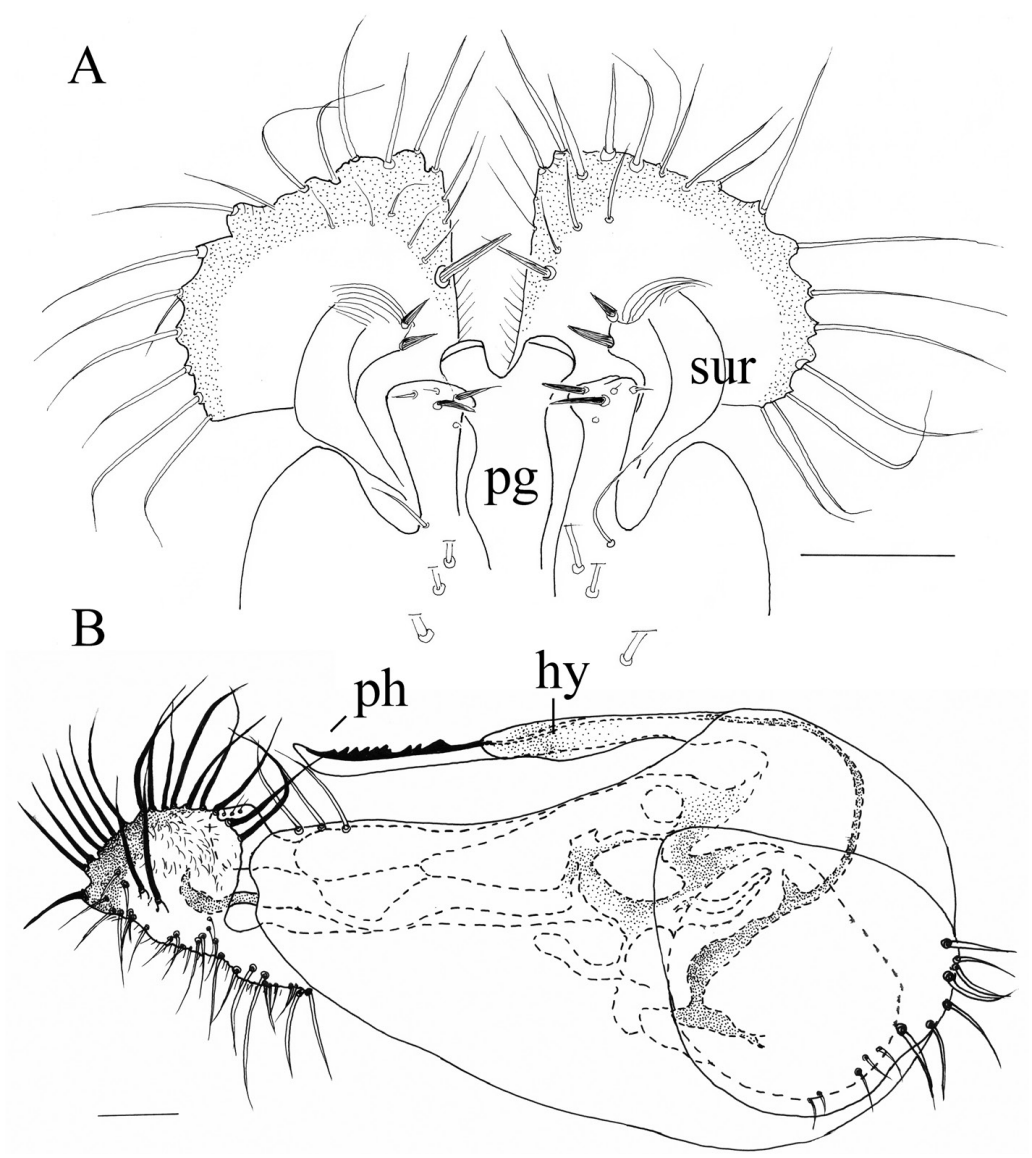
*Lichtwardtiacambodiensis* Tang & Grootaert, sp. n. male terminalia: **A** Cerci, ventral view on inside **B** lateral view male terminalia. Scale bars 0.1 mm.

##### Lichtwardtia dentalis

Taxon classificationAnimaliaDipteraDolichopodidae

Zhang, Masunaga & Yang, 2009

Figs 4 , 5

Lichtwardtia dentalis Zhang, Masunaga & Yang, 2009: 198, figs 1–4.

###### Material examined.

CAMBODIA (all coll. RBINS): 4 males, Siem Reap prov., Angkhor, Preah Khan Temple, 24 January–21 February 2006, Malaise trap in secondary forest (leg. Oul Yothin); 1 male, Siem Reap prov., Angkhor, Preah Khan Temple, 12 May 2006, sweep netting (leg. Oul Yothin).

THAILAND: 1 male (coll. RBINS); Loei prov., Na Haeo Field Research Station (17°29'27.1"N, 101°03'34.6"E), 769 m, 16 May 2003, sweep netting along stream and water fall in secondary forest (leg. P Grootaert).

###### Diagnosis.

**Figure 4.**
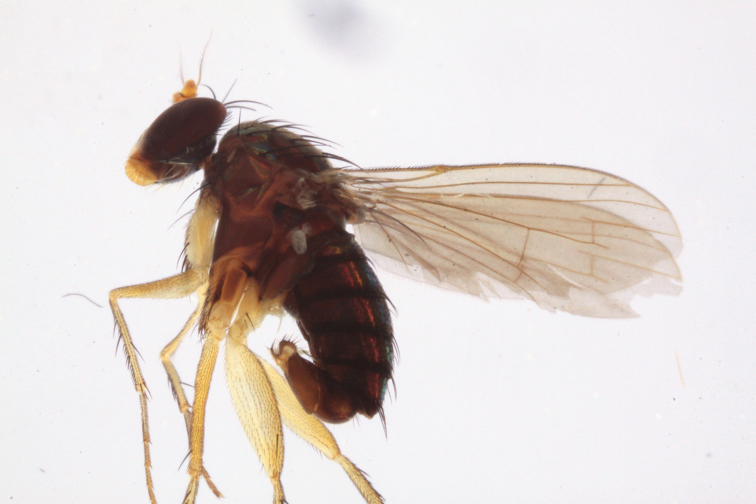
*Lichtwardtiadentalis* Zhang, Masunaga & Yang, 2009 male habitus (Cambodia).

**Figure 5.**
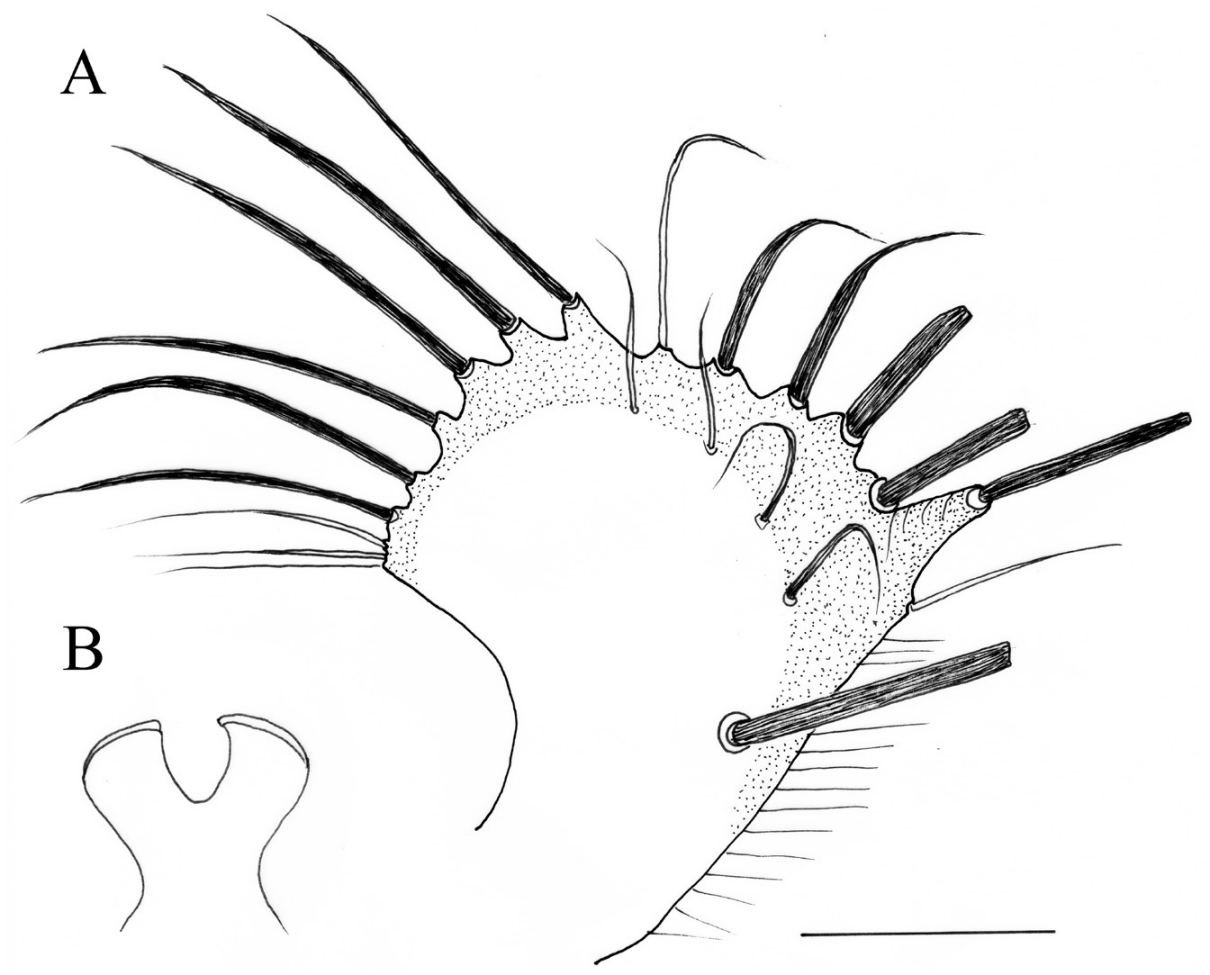
*Lichtwardtiadentalis* Zhang, Masunaga & Yang, 2009 (Cambodia). **A** male cercus **B** tip postgonite. Scale bar: 0.1 mm.

Costa swollen before and at the level where R_1_ joins the costa (Figure 4). Wing clear. Hind coxa yellow. Tip of phallus ventrally with a number of denticles. Hypandrium unarmed. Cercus (Figure 5) with weakly digitated margin. Marginal bristles on cercus brown. Apical marginal bristle longest, blunt-tipped inserted on a long tubercle; subsequent two marginals flattened and blunt-tipped, subsequent bristles with simple tip; 6^th^ bristle fine, pale; subsequent bristles long, brown. A long blunt-tipped interior, longer than the apical marginal.

###### Description.

We refer to the detailed description of *L.dentalis* in Zhang et al. 2009.

###### Comments.

The specimens from northern Thailand and central Cambodia correspond entirely to the description and figures given by Zhang et al. (2009). Especially the widening of the costa where the R_1_ joins the costa as is drawn by Zhang et al. (2009) but not mentioned in the description. The new records are not unlikely since the locality in the Loei province (Thailand) and the one in Cambodia are not so far from the southern Yunnan province (China) that is the type locality of this species.

*L.dentalis* is very closely related to *L.semakau* sp. n. from Singapore and we refer to the comments under the latter species.

###### Distribution.

China, Cambodia, Thailand.

##### Lichtwardtia nodulata

Taxon classificationAnimaliaDipteraDolichopodidae

Grootaert & Tang sp. n.

http://zoobank.org/4E1274BE-F088-4E95-B57A-DC815816C2C3

Figs 6 , 7

###### Material examined.

**Holotype male** (coll. LKCNHM): SINGAPORE: Semakau, 30 August 2012, sweep netting (leg. Jayanthi Puniamoorthy & P Grootaert). **Paratypes** (all coll. LKCNHM): SINGAPORE: 1 male, Pulau Ubin, 14 July 2012, sweep netting in mangrove (leg. Jayanthi Puniamoorthy & P Grootaert); 9 males, 14 females, Kranji nature trail, 27 July 2005, sweep netting in park (leg. P Grootaert); 1 male, Semakau, 13 December 2012, sweep netting in halophilous vegetation on sandy beach (leg. P Grootaert).

###### Diagnosis.

Basal 7/8 of mid coxa and basal half of hind coxa brown, mid coxa with one black band at middle. Vein R_1_ with one oval thickness at where connect to C. Phallus with two rows of black denticles on ventral half, anterior row only with four small sparse denticles, posterior row with eight dense denticles.

###### Description.

**Male.***Body* length 3.6–3.8 mm, wing 3.2–3.3 × 1.0 mm.

*Head* dark metallic green, with thick pale pollinosity; face slightly raised, frons and face both with thick pale pollinosity, gradually narrowed downward. Hairs and bristles on head black except posteroventral hairs pale. Antenna dark yellow; postpedicel nearly triangular, blunt apically, nearly as long as wide, with short black pubescence; arista-like stylus dorsal, inserted at basal half of postpedicel, nearly as long as head width, black, feather-like, with long black pubescence, basal segment 0.3 times as long as apical portion of arista-like stylus. Proboscis brown, with black hairs; palpus dark brown, with one short black apical bristle.

*Thorax* dark metallic green, with pale grey pollinosity. Hairs and bristles on thorax black; five strong dc, ten pairs of acr. Scutellum with two pairs sc, apical pair long strong, basal pair short and weak. Legs mainly dark yellow, but base of fore coxa, basal 7/8 of mid coxa and basal half of hind coxa brown, mid coxa with one black band at middle. Fore coxa anteriorly with three strong bristle at apical half, mid coxa with cluster of anterior hairs three marginal bristles anteriorly and one long strong outer bristle, hind coxa with one long strong outer bristle. Mid trochanter with two weak apical bristle dorsally. Fore femur without distinct bristle. Mid and hind femora each with one preapical pv. Fore tibia with one row of two ad, two pd, one pv and three ap. Mid tibia with three ad, four pd, two pv and four ap. Hind tibia with four ad and three ap. Mid tarsomere I with one short strong pd. Hind tarsomere I with one short strong ad at middle and one av at basal quarter. Relative lengths of tibia and five tarsomeres of legs LI : 6.6 : 3.3 : 1.6 : 1.3 : 1.0 : 1.0; LII : 10.6 : 5.0 : 2.0 : ? : ? : ? (mid tarsus partly lost); LIII : 10.0 : 4.0 : 5.0 : 3.3 : 2.0 : 1.3. Wing nearly hyaline, tinged brown; veins brown. R_1_ with one oval thickness at where connect to C. M with fading M_2_, M_1_ with one short subvein. Crossvein dm-cu somewhat arched. CuAx ratio 1.4. Lower calypter pale with black hairs. Haltere pale.

*Abdomen* metallic green, with pale pollinosity. Hairs and bristles on abdomen black.

*Male genitalia*: Epandrium 1.7 times longer than wide; epandrial lobe with three pale ap. Ventral surstylus and dorsal surstylus both with five short nail-like ap. Cercus nearly triangular, pale except the black ring, with weak digitations around outer margin, with long strong black marginal bristle on digitations, with one long strong bristles on digitation at apical half. Hypandrium simple. Phallus with two rows of black denticles on ventral half, anterior row only with four small sparse denticles, posterior row with eight dense denticles.

**Female.** Similar to male in size and morphology except for the male terminalia.

###### Etymology.

The name refers to the swelling of the costa.

###### Comments.

The new species should be compared to *L.hirsutiseta* since there is a node on the costa that is however distinctly before R_1_ joins the costa (Figure 22) in the latter.

###### Distribution.

Singapore.

**Figure 6.**
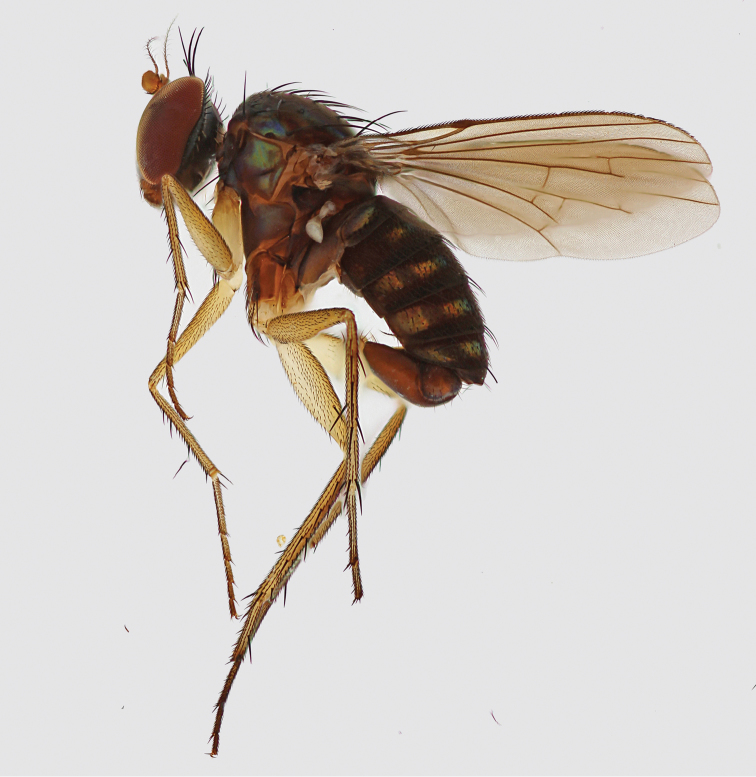
*Lichtwardtianodulata* Grootaert & Tang, sp. n. Holotype male habitus.

**Figure 7.**
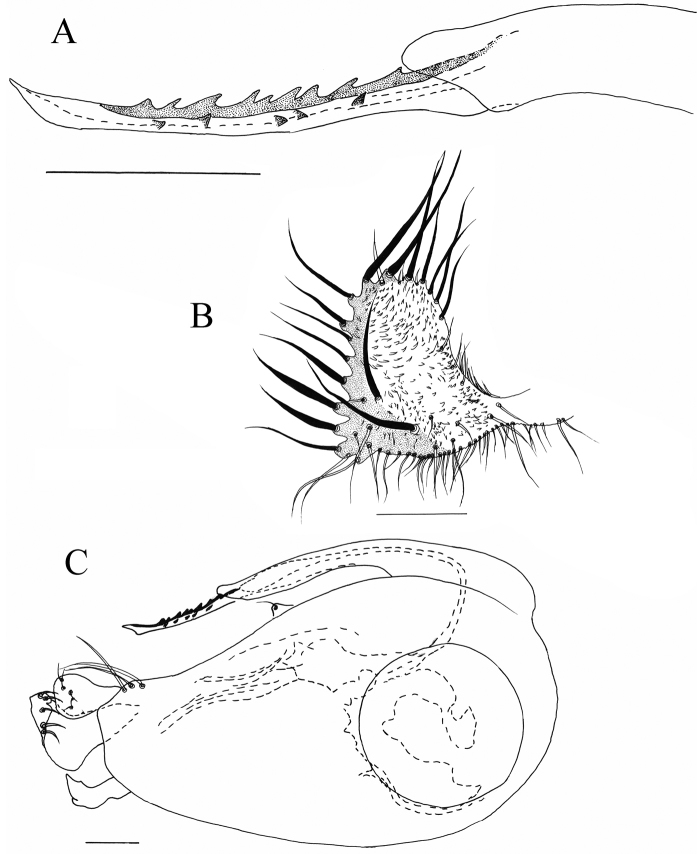
*Lichtwardtianodulata* Grootaert & Tang, sp. n. Male terminalia: **A** tip of phallus **B** epandrium lateral **C** cercus inside view. Scale bars 0.1 mm.

##### Lichtwardtia semakau

Taxon classificationAnimaliaDipteraDolichopodidae

Grootaert & Tang sp. n.

http://zoobank.org/36C23F6C-8675-45A1-8860-8271B32C4088

Figs 8 , 9

###### Material examined.

**Holotype male** (coll. LKCNHM): SINGAPORE: Semakau, back mangrove in the old mangrove (1°12'19.9"N, 103°45'34.1"E), 3 April 2012, sweep netting along path (leg. P Grootaert). **Paratype** (coll. LKCNHM): 1 female, same provenance as holotype.

###### Diagnosis.

Antenna entirely dark yellow. Arista-like stylus feathered. Wing clear, faintly brownish tinged. No thickening of the costa where R_1_ joins the costa. Fore and hind coxae entirely yellow. Mid coxa with a rectangular brown spot anteriorly, posteriorly pale brownish. Cercus yellow, brownish seamed with margin distinctly digitated and bearing five thickened marginal bristles near the tip. Hypandrium simple, lacking protuberances. Phallus ventrally with at least twelve black denticles.

###### Description.

**Male.***Body* length 3.9 mm, wing 3.5 × 1.2 mm. Head dark metallic green, with thick pale pollinosity; face slightly raised, frons and face both with thick pale pollinosity, gradually narrowed downward. Hairs and bristles on head black but lower postocular hairs pale. Antenna dark yellow (Figure 21); postpedicel nearly triangular, blunt apically, 1.2 times as long as wide; arista-like stylus black, nearly as long as width of head, feather-like, with long pubescence, basal portion 0.3 times as long as apical portion. Proboscis dark yellow, with black hairs; palpus dark yellow, with a short black apical bristle.

*Thorax* dark green, with pale grey pollinosity. Hairs and bristles on thorax black; five strong dc, ten pairs of long acr. Scutellum with two pairs sc, apical pair long strong, basal pair short and weak. Legs mainly yellow, but mid coxa with a dark brown rectangular spot anteriorly and a pale brown posteriorly. Fore and mid coxae anteriorly with rows of bristle-like hairs, fore and mid coxae anteriorly with three long strong preapical bristles and rows of anterior bristles, mid coxa with one outer bristles at apical third, hind coxa with two outer bristles, basal one strong, apical one relatively weak. Mid trochanter with two ap dorsally. Hind trochanter with one outer bristle at middle. Fore femur with one strong pv at middle. Mid femur with one strong pd and one preapical pv. Hind femur with one strong pd at apical quarter. Fore tibia with one ad, three pd, one av and three ap. Mid tibia with two ad, three pd, one pv and four ap. Hind tibia with three ad, three pd (one preapical), two weak pv and three ap. Fore tarsomere I with one short av at base. Hind tarsomere I with one strong ad at middle and two short apical bristle. Relative lengths of tibia and five tarsomeres of legs LI : 7.0 : 3.2 : 1.2 : 1.2 : 1.0 : 1.0; LII : 6.0 : 4.0 : ? : ? : ? : ? (mid tarsus partly lost); LIII : 10.4 : 4.0 : ? : ? : ? : ?. Wing nearly hyaline, nearly pale; veins brown. No thickening of the costa where R_1_ joins the costa. M with fading M_2_, M_1_ with one short subvein. Crossvein dm-cu straight. CuAx ratio 1.2. Lower calypter pale with black hairs. Haltere pale.

*Abdomen* metallic green, with pale pollinosity. Hairs and bristles on abdomen black.

Male terminalia (Figure 9): Epandrium 1.8 times longer than wide; ventral epandrial margin at the level of the reduced epandrial lobe with three long pale bristles (Figure 9D), and with a small basoventral bristle. Ventral surstylus and dorsal surstylus both with five short nail-like ap. Cercus nearly triangular, pale except the black margin, with distinct digitations around outer margin, bearing strong black marginal bristles. On the inside a single strong bristle near the dorsal margin, with two relatively strong blade-like bristles on digitations at apical half. Hypandrium simple. Phallus with black small dense irregular denticles on ventral half (Figure 9A).

**Female.** Body length 4.3 mm, wing 3.4 × 1.3 mm. Resembling the male except for the terminalia and face wider.

###### Etymology.

The name refers to the type locality Semakau, an island on the southern coast of Singapore.

###### Comments.

*L.semakau* sp. n. resembles very much *L.dentalis*Zhang et al. 2009 described from the Yunnan province (China). It differs in that *L.dentalis* has a broadening of the costa at the level where the R_1_ joins the costa (Zhang et al. 2009, Figure 1). *L.semakau* sp. n. has no swelling at all of the costa. The outer margin of the cercus seems more weakly digitated with only a strong marginal bristle dorsally and one on the tip. In *L.semakau* sp. n., the margin of the cercus is more deeply indented and there are five broad black marginal bristles at the tip of the cercus.

*L.semakau* sp. n. should be compared also to *L.cambodiensis* sp. n. that differs in the male also by a thickening of a costa and the marginal bristles on the cercus that are all thin and paler.

###### Distribution.

Singapore.

**Figure 8.**
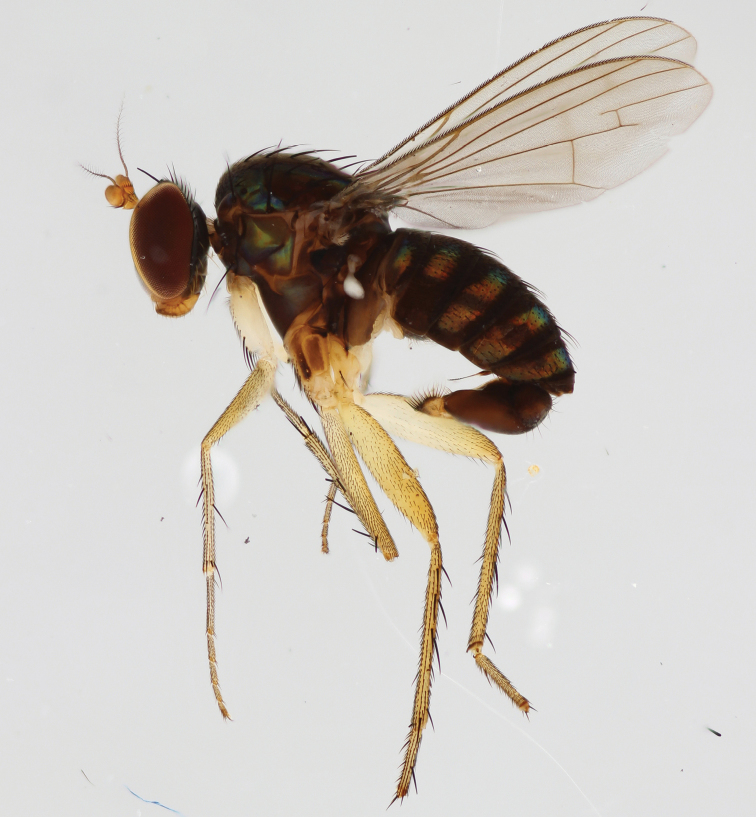
*Lichtwardtiasemakau* Grootaert & Tang, sp. n. (Singapore).

**Figure 9.**
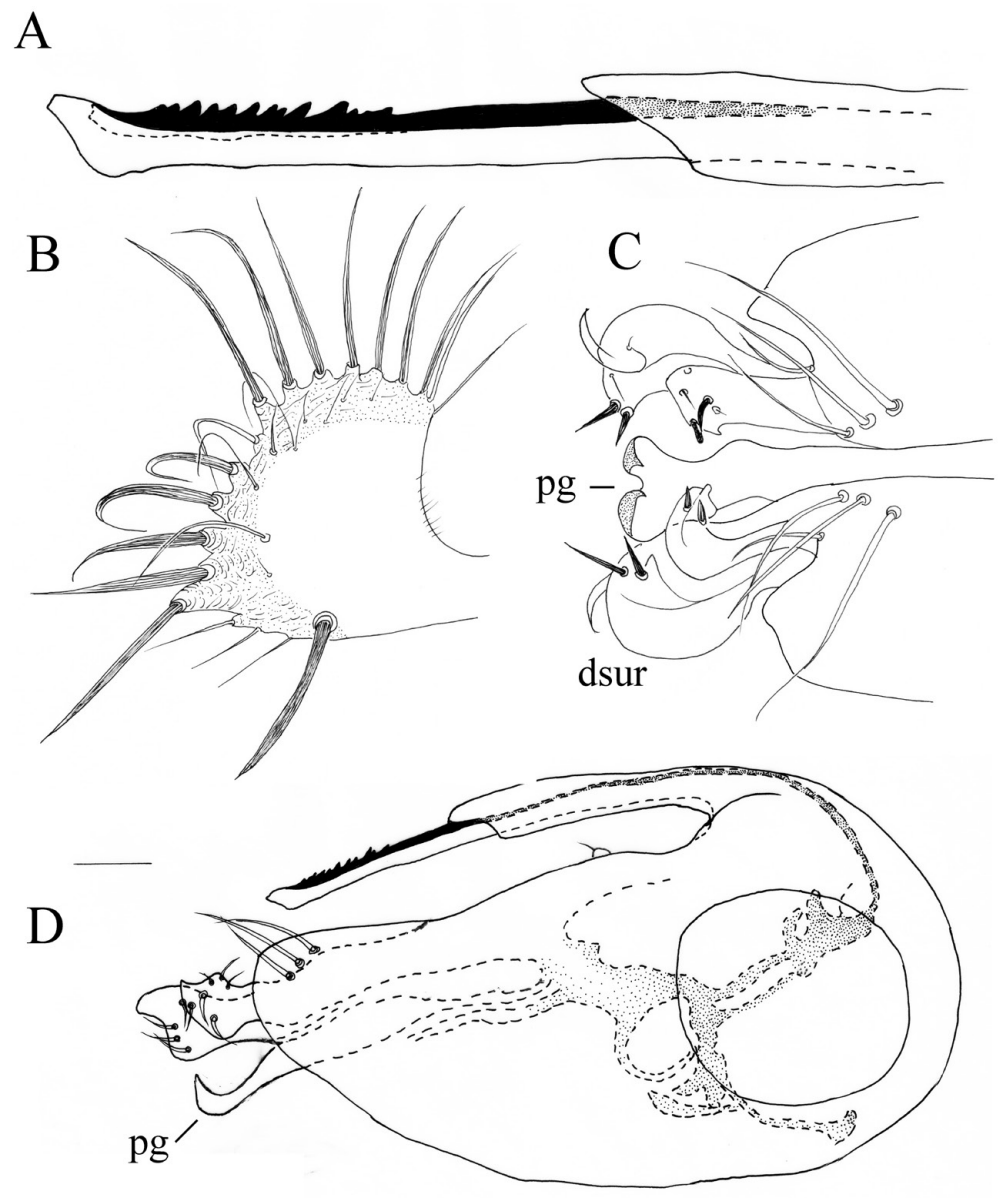
*Lichtwardtiasemakau* Grootaert & Tang, sp. n. male terminalia (Singapore): **A** tip phallus **B** cercus **C** surstyli ventral view **D** epandrium lateral. Scale bar: 0.1 mm.

##### Lichtwardtia singaporensis

Taxon classificationAnimaliaDipteraDolichopodidae

Grootaert & Tang sp. n.

http://zoobank.org/7D25B148-FAC6-4EB7-B1B0-5162751376EF

Figs 10 , 11

###### Material examined.

**Holotype male** (coll. LKCNHM): SINGAPORE: West Coast Park, 7 December 2003, shrubs along sandy beach (leg. P Grootaert), sweeping. **Paratype** (coll. LKCNHM): 1 female; collecting information same to the holotype

###### Diagnosis.

Wings clear, but having the cross veins brownish seamed. Mid coxa brown with one anterior dark brown stripe and one lateral band, both dark brown. Hind coxa yellow. Hypandrium simple. Phallus with one row of five big clear regular black denticles ventrally, with one blunt denticle hidden in hypandrium.

###### Description.

**Male.***Body* length 3.7 mm, wing 3.4 × 1.1 mm.

*Head* dark metallic green, with thick pale pollinosity; face slightly raised, frons and face both with thick pale pollinosity, gradually narrowed downward. Hairs and bristles on head black except posteroventral hairs pale. Antenna dark yellow; postpedicel nearly triangular, blunt apically, 1.2 times as long as wide, with pale pubescence; arista-like stylus dorsal, inserted at basal half of postpedicel, nearly as long as head width, dark yellow, feather-like, with long black pubescence, basal segment 0.3 times as long as apical portion of arista-like stylus. Proboscis dark yellow, with black hairs; palpus dark yellow, with one black apical bristle.

*Thorax* dark green, with pale grey pollinosity. Hairs and bristles on thorax black; five strong dc, ten pairs of strong acr. Scutellum with two pairs sc, apical pair long strong, basal pair short and weak. Legs mainly yellow, but fore coxa somewhat brownish at base, mid coxa brown with one anterior dark brown stripe and one lateral band, both dark brown, hind coxae dark yellow, lightly brownish at basal half; mid and hind tarsi brown from tip of tarsomere II to tarsomere V. Fore and mid coxae anteriorly with row of thin bristles and four strong bristles at apical half, hind coxa with two short outer bristles. Fore trochanter with three weak outer bristle at middle. Mid trochanter with two short weak outer bristles. Fore femur with one short weak av. Mid femur with one strong pd at apical 1/6 and one preapical pv. Hind femur with one strong pd at apical quarter. Fore tibia with two pd, one av, and one pv and three short ap. Mid tibia with two ad, four pd (basal 1^st^ relatively weak), one pv at middle and four ap. Hind tibia with four ad, four pd, two pv and three ap. Fore tarsomere I with one short av at base. Hind tarsomere I with one short strong ad at middle, one pv at basal fifth and two short apical bristle. Relative lengths of tibia and five tarsomeres of legs LI : 6.0 : 3.0 : 1.6 : 1.2 : 1.0 : 1.0; LII : 8.0 : 5.0 : 2.0 : 1.6 : 1.2 : 1.0; LIII : 10.0 : 4.0 : 4.0 : 3.0 : 2.0 : 1.4. Wing nearly hyaline, cross veins seamed brownish; veins brown. M with fading M_2_, M_1_ with one short subvein. Crossvein dm-cu straight. CuAx ratio 1.0. Lower calypter pale with black hairs. Haltere pale.

*Abdomen* metallic green, with pale pollinosity. Hairs and bristles on abdomen black.

*Male terminalia*: Epandrium 1.8 times longer than wide; epandrial lobe with three pale ap. Surstylus with five short nail-like ap. Cercus nearly triangular, pale, covered by thin pale bristles, with weak digitations around outer margin, with long strong pale marginal bristle on digitations. Hypandrium simple. Phallus with one row of five big clear regular black denticles ventrally, with one blunt denticle hidden in hypandrium.

**Female.** Body length 3.6–4.0 mm, wing length 3.1–3.2 × 1.1 mm. Very similar to male, but postpedicel as long as wide. Wing hyaline with anterior border faintly brownish and cross veins brownish seamed. No swelling of the costa before or at the point where R_1_ joins the costa. The ratio of the proximal section of M_1_, and the distal section is 0.4/0.6 (Figure 12). Thus the distal section is much longer than the proximal section. Fore coxa yellow, mid coxa brown and hind coxa yellowish.

###### Comments.

This species is comparable to *L.infuscata* sp. n. but the latter has quite brownish wings also with the cross veins seamed brown. Both species have also the hind coxa entirely yellow and the phallus bears ventrally rather strong spines. In *L.singaporensis* sp. n. there are five strong spines while in *L.infuscata* there is a double row of ten denticles that are smaller than in *L.singaporensis* sp. n. The epandrium has a pointed tip while its tip in *L.infuscata* sp. n. is truncated. The size of the cercus is larger in *L.infuscata* sp. n. *L.infuscata* has a pale ventral protuberance with a black seam on the epandrium on which the phallus-hypandrial complex is resting. This protuberance is absent in *L.singaporensis* sp. n. The brownish seams along the cross veins suggest that *L.singaporensis* sp. n. represents the enigmatic *L.ziczac*. However we refrain from given the description of the male of *L.singaporensis* sp. n. as the true *L.ziczac*. See comments under *L.ziczac*.

###### Distribution.

Singapore.

**Figure 10.**
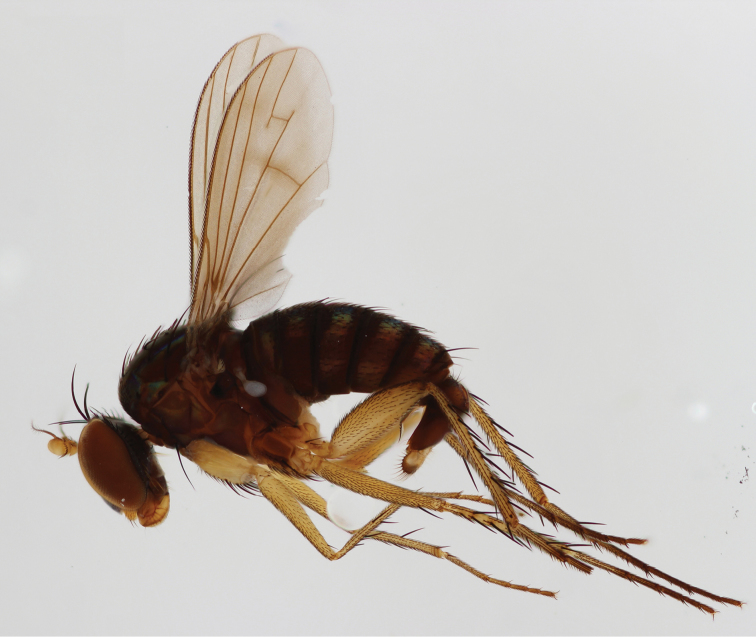
*Lichtwardtiasingaporensis* Grootaert & Tang, sp. n.: Habitus male.

**Figure 11.**
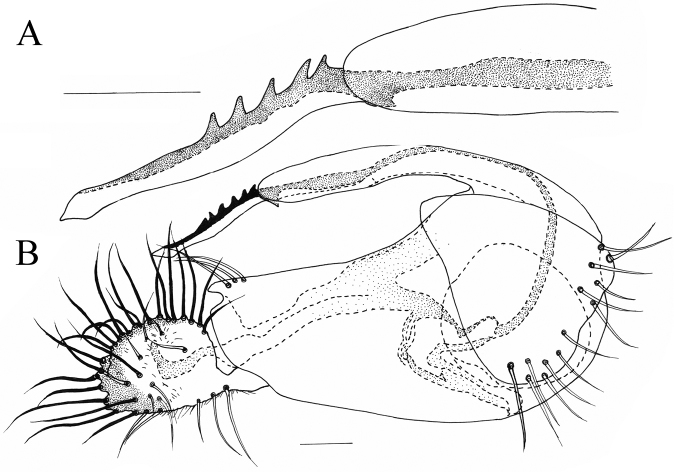
*Lichtwardtiasingaporensis* Grootaert & Tang, sp. n. male terminalia (Singapore):). Male terminalia: **A** Tip phallus **B** epandrium lateral. Scale bars: 0.1 mm.

##### Lichtwardtia conspicabilis

Taxon classificationAnimaliaDipteraDolichopodidae

group

###### Diagnosis.

The hypandrium has a bifurcated tip and a curved appendage. The phallus has a preapical ventral process.

##### Lichtwardtia conspicabilis

Taxon classificationAnimaliaDipteraDolichopodidae

Tang & Grootaert sp. n.

http://zoobank.org/EB34AB9B-242C-4698-80AA-DD536C6B70D1

Figs 12 , 13

###### Material examined.

**Holotype male** (coll. RBINS): CAMBODIA: Siem Reap prov., Angkhor, Preah Khan Temple, 6 June 2006; sweep netting (leg. Oul Yothin). **Paratypes** (coll. RBINS): CAMBODIA: 2 males, locality same to the holotype, 4–14 April 2006, Malaise trap (leg. Oul Yothin).

###### Diagnosis.

Antenna nearly entirely dark yellow. Wing brownish tinged, a distinct swelling of the costa present just before R_1_ joins the costa. Mid coxa with two light brown bands laterally. Hind coxa entirely yellow. Cercus with elongate tip bearing a longer bristle than the other marginals; hypandrium with bifurcate tip and a black curved appendage on the right-hand side. Phallus wide, with preapical ventral process.

###### Description.

**Male.***Body* length 3.7 mm, wing 3.5 × 1.1 mm.

*Head* dark metallic green, with thick pale pollinosity; face slightly raised, frons and face both with thick pale pollinosity, gradually narrowed downward. Hairs and bristles on head black; upper four postocular bristles black but all lower postoculars pale. Antenna dark yellow except portion at level of arista-like stylus slightly brownish. Postpedicel nearly triangular, blunt apically, with brown pubescence, nearly as long as wide; arista-like stylus dark brown, nearly as long as width of head, feather-like, with long pubescence, basal portion 0.2 times as long as apical portion. Proboscis dark yellow, with black hairs; palpus dark yellow, with one short weak black apical bristle.

*Thorax* dark green, with pale grey pollinosity. Hairs and bristles on thorax black; five strong dc, ten pairs of long acr. Scutellum with two pairs sc, apical pair long and strong, basal pair very short and weak. Legs mainly yellow, but mid coxa with two light brown bands laterally, hind coxa entirely yellow. Fore and mid coxae anteriorly to laterally with rows of bristle-like hairs and six marginal bristles; mid coxa with one outer bristles at apical third, hind coxa with two outer bristles, basal one strong, apical one weak. Mid trochanter with two ap. Hind trochanter with one outer hair at middle. Fore femur without distinct bristles. Mid femur with one strong ad and one preapical pv. Hind femur with one strong anterior on apical fourth. Fore tibia with three ad, one av and three ap (of which one posterodorsal very long). Mid tibia with two ad, five pd, two pv, and four ap. Hind tibia with three ad, two pd, one pv and four ap. Fore tarsomere I with one short av at base. Hind tarsomere I with one strong dorsal beyond middle, two short av close to base and two short apical bristle. Relative lengths of tibia and five tarsomeres of legs LI : 10.0 : 4.0 : 2.5 : 2.0 : 1.5 : 1.5; LII : 16.0 : 7.5 : 4.0 : 3.0 : 1.5 : 1.0; LIII : 18.0 : 7.5 : 7.5 : 6.0 : 4.0 : 2.5. Wing brownish tinged, veins brown. M with fading M_2_, M_1_ with one short subvein. Crossvein dm-cu straight. CuAx ratio 1.3. Lower calypter pale with long black hairs. Haltere white.

*Abdomen* metallic green, with pale pollinosity. Hairs and bristles on abdomen black.

*Male terminalia* (Figure 13): Epandrium 1.6 times longer than wide. Surstylus with several digitations. Cercus ovoid with short apical process, pale except the black seam around the apical margin, with weak digitations around outer margin only, ventral marginal bristles black; with elongate tip bearing a longer bristle than the other marginal, a long black inner bristle below the tip. Hypandrium thick, with bifurcate tip and a black curved appendage on the right-hand side. Phallus wide, with preapical ventral process.

**Female**. Unknown.

###### Comments.

This species resembles superficially *L.polychroma* in having a swelling of the costa and by the similar elongate cercus and the complicated structure of the tip of the hypandrium. However, *L.polychroma* possesses a large brown tooth at the right side. *L.conspicabilis* sp. n. has a curved black extension of the left side and the tip of the phallus is deeply indented.

###### Etymology.

The name *conspicabilis* refers to the remarkable structure of the hypandrium.

###### Distribution.

Cambodia.

**Figure 12.**
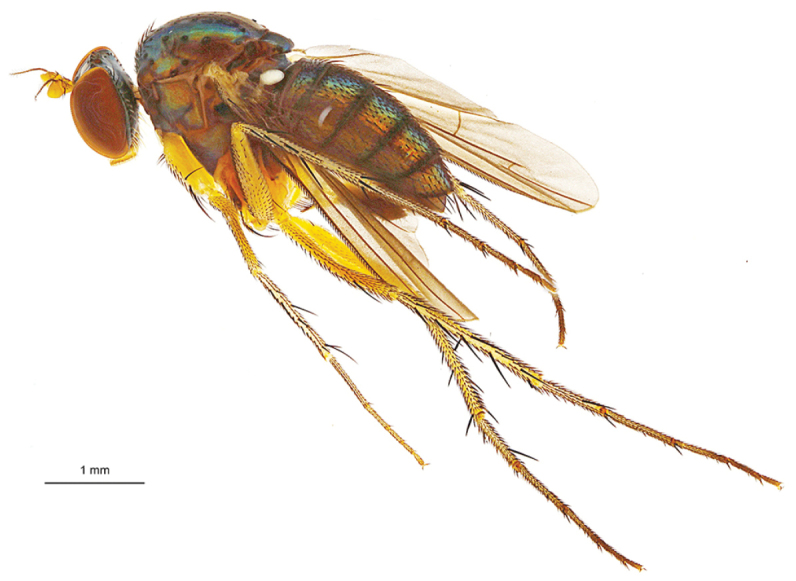
*Lichtwardtiaconspicabilis* Tang & Grootaert, sp. n. male habitus. *Lichtwardtiainfuscata* Tang & Grootaert, sp. n. Male terminalia: **A** Phallus **B** epandrium lateral view. Scale bar: 0.1 mm.

**Figure 13.**
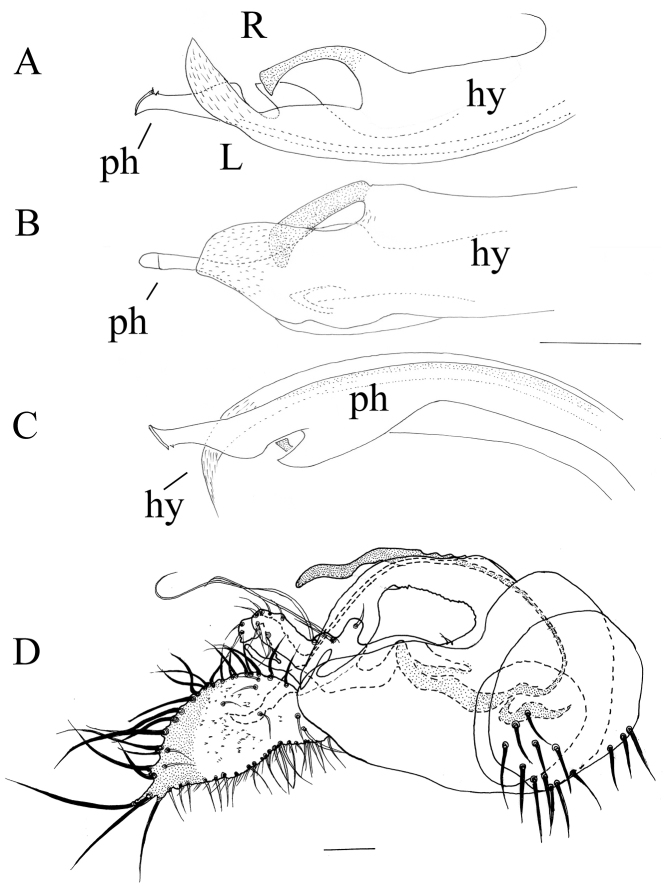
*Lichtwardtiaconspicabilis* Tang & Grootaert, sp. n. male terminalia: **A** right view on hypandrium and phallus **B** ventral view of hypandrium and phallus **C** view on left side of phallus and hypandrium **D** lateral view of hypopygium. Scale bars: 0.1 mm.

##### Lichtwardtia monstruosa

Taxon classificationAnimaliaDipteraDolichopodidae

Tang & Grootaert sp. n.

http://zoobank.org/D6EBAA51-43A7-4803-9CB9-84CF23DEA3CA

Figs 14 , 15

###### Material examined.

**Holotype male** (coll. RBINS): CAMBODIA: Siem Reap prov., Angkhor, Preah Khan Temple, 18–25 April 2006, Malaise trap in secondary forest (leg. Oul Yothin). **Paratype** (coll. RBINS): 1 male, same provenance as holotype.

###### Diagnosis.

Postpedicel of antenna mostly brownish yellow. Wing faintly tinged brownish, no swelling of the costa present. Mid coxa with two brown bands laterally, one brown, one light brown. Hind coxa entirely yellow. Tip of hypandrium bifurcate with a black narrow ventral arm that is strongly toothed and a broad arm dorsally yellowish at base and black at tip; apex rounded, bordered with teeth. Phallus yellowish with a very strong yellowish brown dorsal hook near middle resting in the ventral cavity of the epandrium; apical half of phallus, with a ventral curved followed by a strong dorsal curved; tip pointed.

###### Description.

**Male.***Body* length 4.0 mm, wing 3.8 × 1.3 mm. Head with frons shining metallic green; face slightly raised, as wide as postpedicel, frons and face covered with a thick white dusting. Hairs and bristles on head black; upper five postoculars black, lower postocular bristles pale. Antenna mainly yellow, postpedicel largely brownish yellow; postpedicel nearly triangular, blunt apically, with yellow pubescence, 1.2 times as long as wide; arista-like stylus dark yellow, nearly as long as width of head, feather-like, basal portion 0.5 times as long as apical portion. Proboscis dark yellow, with black hairs; palpus dark yellow, with one short weak black apical bristle.

*Thorax* dark metallic green, with a fine pale grey pollinosity. Hairs and bristles on thorax black; five strong dc, ten pairs of acr half as long as dc. Scutellum with two pairs sc, apical pair long strong, basal pair short and weak. Legs mainly yellow, but mid coxa with two brown bands laterally, one brown, one light brown. Fore and mid coxae anteriorly to laterally with rows of bristle-like hairs and eight preapical bristles; mid coxa with one strong outer bristles at apical third, hind coxa with two outer bristles, basal one strong, apical one relatively weak. Mid trochanter with two dorsal ap. Hind trochanter with one outer bristle at middle. Fore femur without distinct bristles. Mid femur with one strong ad and a weak pd. Hind femur with one strong ad at apical quarter. Fore tibia with two ad, two pd, one av and four ap. Mid tibia with two ad, three pd, one pv and four ap. Hind tibia with four ad, four d and four ap; ventrally in basal third with a row of small erect hairs. Fore and mid tarsomere I without distinct bristles. Hind tarsomere I with one strong d at apical third. Relative lengths of tibia and five tarsomeres of legs LI : 8.2 : 5.0 : 2.0 : 1.6 : 1.0 : 1.0; LII : 13.2 : 6.6 : 3.3 : 2.6 : 2.0 : 1.3; LIII : 13.2 : 6.0 : 6.0 : 4.3 : 3.3 : 1.6. Wing nearly hyaline, tinged brown; veins brown. M with fading M_2_, M_1_ with one short subvein. Crossvein dm-cu straight. CuAx ratio 1.0. Lower calypter pale with black hairs. Haltere pale.

*Abdomen* metallic green, with fine pale pollinosity. Hairs and bristles on abdomen black.

**Figure 14.**
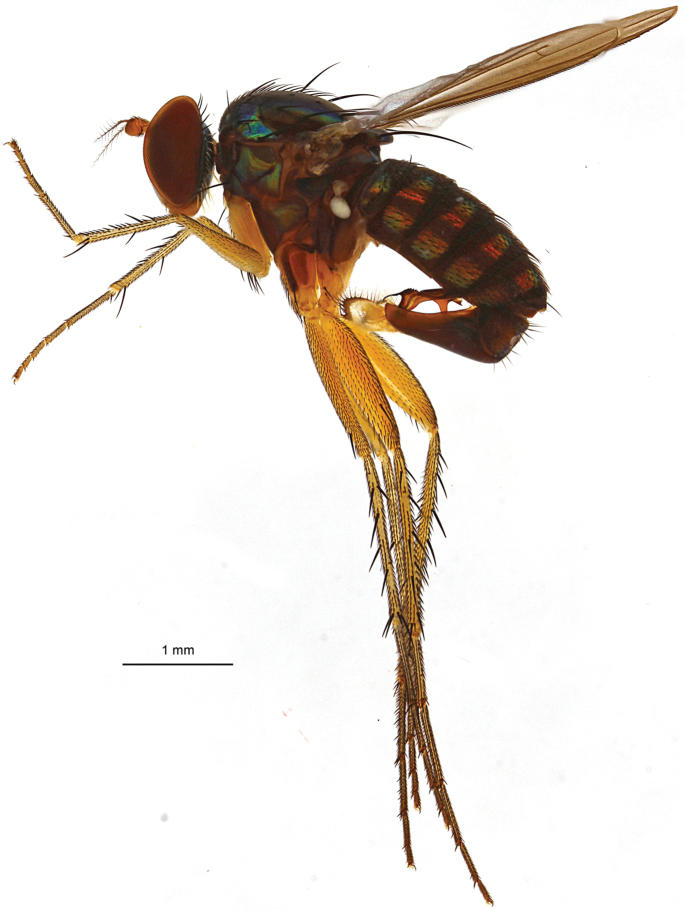
*Lichtwardtiamonstruosa* Tang & Grootaert, sp. n. (Cambodia).

**Figure 15.**
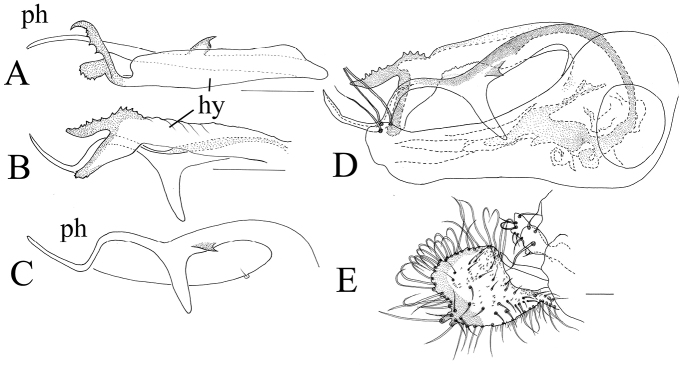
*Lichtwardtiamonstruosa* Tang & Grootaert, sp. n. Male terminalia (Cambodia): **A** hypandrium ventrally **B** hypandrium lateral **C** phallus lateral **D** epandrium lateral **E** cercus lateral. Scale bars: 0.1 mm.

*Male terminalia* (Figure 15): Epandrium 2.1 times longer than wide, narrowing towards tip; epandrial lobe with three long pale bristles. Ventral surstylus with three long pale bristles. Cercus nearly quadrate, as long as wide, pale except the black marginal seam, with weak digitations around outer margin, with black marginal bristles on digitations. Tip of hypandrium bifurcate with a black narrow ventral arm that is strongly toothed and a broad arm dorsally yellowish at base and black at tip. Apex rounded bordered with teeth. Phallus yellowish with a very strong yellowish brown dorsal hook near middle resting in the ventral cavity of the epandrium (Figure 14); a short black pointed tooth at the left side and at the base of the large tooth; apical half of phallus, with a ventral curved one followed by a strong dorsal curved; tip pointed.

**Female**. Unknown.

###### Etymology.

The species name alludes to the monstrous appendages on the male terminalia.

###### Comments.

The male is easily recognised having these huge extensions on the terminalia. The very strong dorsal tooth on the phallus resembles superficially the strong tooth present on the tip of the hypandrium of *L.polychroma* and *L.zhangae*. However the origin of these structure is different: in *L.monstruosa* sp. n., it is on the phallus, while in the others it is on the hypandrium.

###### Distribution.

Cambodia.

##### Lichtwardtia polychroma

Taxon classificationAnimaliaDipteraDolichopodidae

-group

###### Diagnosis.

The cercus is more or less triangular, with some strong flattened marginals and a dorsal marginal that shifted to the inside. The postgonite is tubiform. The tip of the phallus has no denticle. The hypandrium has a strong subapical dorsal hook.

##### Lichtwardtia polychroma

Taxon classificationAnimaliaDipteraDolichopodidae

(Loew, 1864)

Figs 16 , 17 , 18

Rhagoneurus polychromus Loew, 1864: 346, Fig. 3, a, b, c. Male & female. Type locality: Sri Lanka.

###### Material examined.

**Figure 16.**
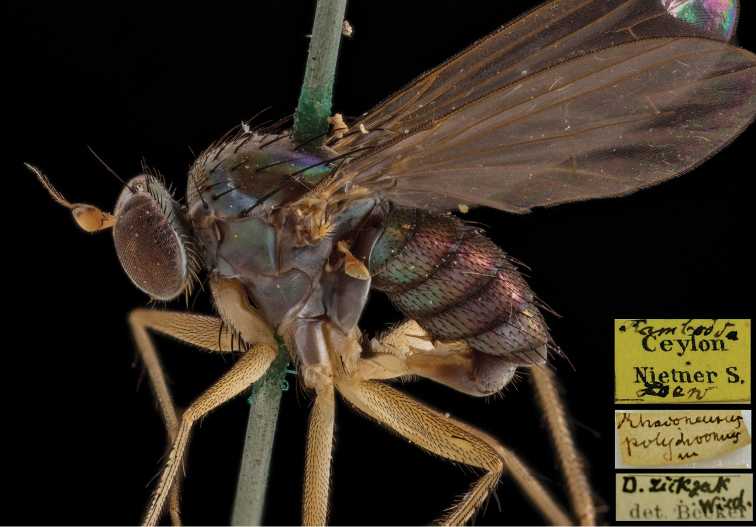
*Lichtwardtiapolychroma* (Loew, 1864) lectotype male habitus, Sri Lanka (photograph by Bernhard Schurian).

There is a single male conserved in the collection of Becker (MfN, Berlin) bearing the label “*Rhaconeuruspolydromus* m” in the handwriting of Loew (Figure 16). We think that the “m” stands for mihi (mine or my species) or for manuscript name. It was this specimen that Becker cited in his 1922 book commenting on the writing error *Rhaconeurus* by Loew which should have been *Rhagoneurus*. Becker labelled the specimen *zickzak* Wied. det. Becker though he published it as *zickzack*. It bears a third yellow label with “Ceylon Nietner S.” in print and in handwriting Rambodda. Below we see in handwriting Loew (Figure 16). Nietner S. means Nietner sammelt.

The information on the locality is new because Loew did not give a precise locality in his description. Rambodda (nowadays cited as Ramboda) is a small village in Sri Lanka known for its famous waterfalls. Johannes (John) Nietner (died 1874) was a German naturalist chiefly interested in botany and entomology. Born in Potsdam, he was a plantation owner in Rambodda, Ceylon and described many new insect species from the island. Having a special interest in insects, he made large collections and sent specimens for study by experts abroad. Collections from him are in the Deutschen Entomologischen Institut, the Museum für Naturkunde in Berlin, in the Naturhistorischen Museum in Vienna and the Natural History Museum in London.

We designate the male as lectotype since Loew did not designate a holotype. A female was included in the description but we failed to find it. Stacked images of the lectotype male were provided by the courtesy of Mr. Bernhard Schurian and Sven Marotske (MfN, Berlin).

###### Comments.

Zhang et al. 2009 re-described and illustrated a similar species as *Lichtwardtiaziczac* (Wiedemann). Thanks to her detailed drawings and re-description we could see that her species does not correspond to the female holotype of *L.ziczac*. The latter has the cross veins brownish seamed (Figure 25). The male that she studied was found on Bali (Indonesia) and we are not sure if it is really conspecific with our *L.polychroma* from Cambodia although the huge dorsal hook near the tip suggests so. Temporarily we consider the specimens from Cambodia as *L.polychroma* both having a swelling of the costa, while the species from Bali without swelling of the costa as a different new species.

###### Additional material examined for the descriptions.

CAMBODIA (all coll. RBINS): 1 male, Siem Reap prov., Angkhor, Preah Khan Temple; 17–24 February 2005, Malaise trap in secondary forest (leg. Oul Yothin). 4 males; same provenance, 24 January–21 February 2006. 1 male, same provenance, 28 March–7 April 2006. 1 male; Siem Reap prov., Angkhor, Bakheng; 23–31 October 2005; Malaise trap in secondary forest (leg. Oul Yothin).

###### Diagnosis.

**Figure 17.**
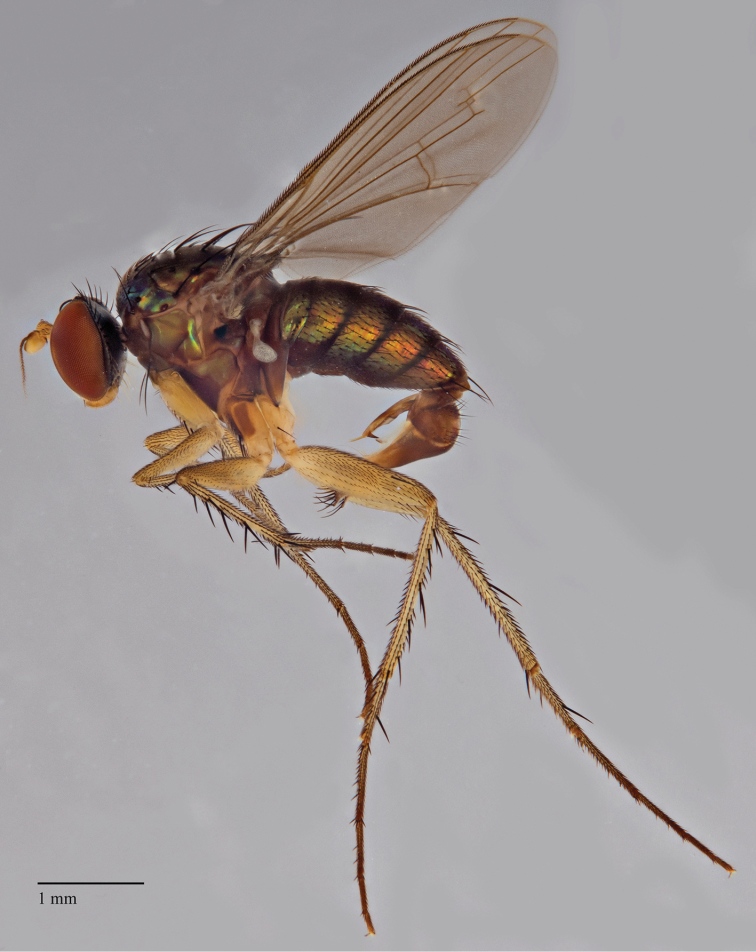
*Lichtwardtiapolychroma* (Loew, 1864) male habitus Cambodia (photograph Maimon Hussin).

**Figure 18.**
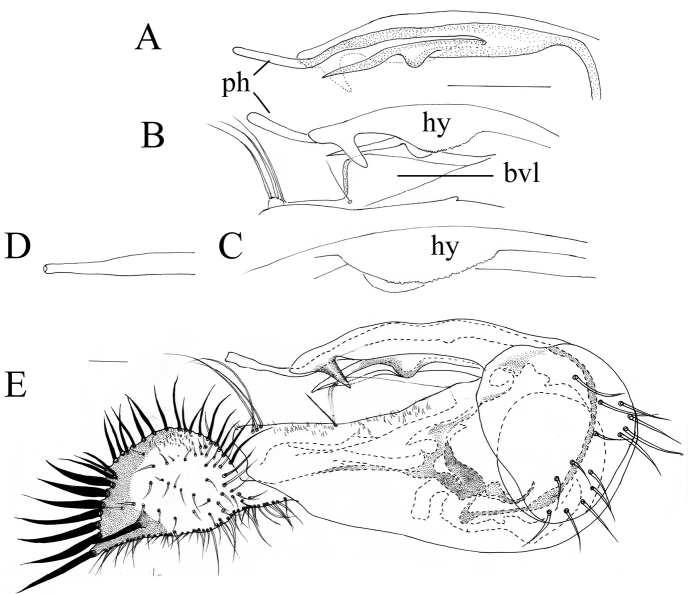
*Lichtwardtiapolychroma* (Loew, 1864) male terminalia (Cambodia): **A** phallus **B** hypandrium covering phallus **C** hypandrium with toothed dorsal border **D** tip postgonite **E** epandrium lateral view, but cercus inner view. Scale bars: 0.1 mm.

Antenna largely yellow, legs yellow (Figs 16, 17). Postpedicel 1.5 times as long as wide. Arista-like stylus with rather short hairs. Wing entirely hyaline with a short widening of the costa just before R_1_ joins the costa. Mid coxa anteriorly with a blackish brown band, posteriorly with a brown band. Hind coxa entirely yellow. Hypandrium (Figure 18) with a strong brown subapical spine. Phallus smooth. Cercus pointed with broadened bristles.

###### Description.

**Male.***Body* length 4.2 mm, wing 3.8 × 1.3 mm.

*Head* dark metallic green, with thick pale pollinosity; face slightly raised, frons and face both with thick pale pollinosity, gradually narrowed downward. Hairs and bristles on head black except postocular bristles yellow. Antenna yellow; postpedicel with extreme tip and dorsal margin brownish; elongate triangular, blunt at tip, nearly as long as wide; arista-like stylus with long densely set hairs. Proboscis dark yellow, with short black hairs; palpus, dark yellow with one black apical bristle.

*Thorax* dark green, with pale grey pollinosity. Hairs and bristles on thorax black; five strong dc, ten pairs of strong acr, with dense short strong bristles at anterior portion. Scutellum with two pairs sc, apical pair long strong, basal pair short and weak. Legs mainly yellow. Fore and hind coxa entirely yellow, but mid coxa with a black band anteriorly and a broad band posteriorly. Fore coxa anteriorly at base with a few short erect bristles, anteriorly densely covered with short black bristle-like hairs, four very long ap and a few shorter bristles. Mid coxa anteriorly densely covered with short black hairs, with a long outer bristle at the tip of the blackish band; hind coxa with two outer bristles, basal one strong, apical one short and weak. Mid and hind trochanters both with several short weak hairs. Fore femur lacking ventral bristles. Mid femur with one preapical pv. Hind femur with one strong ad at apical quarter. Fore tibia with two ad, two pd, one av, and three ap. Mid tibia with two ad, three pd, one av, and four ap; all long strong. Hind tibia with two ad, four pd, one pv, and three ap; all long. Hind tarsomere I with one strong ad at middle, one short strong ad at basal third and two short apical bristle. Relative lengths of tibia and five tarsomeres of legs LI : 9.0 : 6.0 : 2.4 : 1.2 : 1.0 : 1.0; LII : 16.0 : 8.0 : 5.0 : 4.0 : 2.4 : 1.6; LIII : 18.0 : 6.0 : 4.0 : 4.0 : ? : ?. Wing nearly hyaline, tinged brownish, veins brown. M with fading M_2_, M_1_ with one short subvein. Crossvein dm-cu straight. CuAx ratio 1.1. Lower calypter pale with black hairs. Haltere pale.

*Abdomen* metallic green, with pale pollinosity. Hairs and bristles on abdomen black.

*Male terminalia*. Epandrium 1.9 times longer than wide (Figure 18E); epandrial lobe with three long pale ap. Surstylus thin and long with three thin ap and three bristles at middle. Cercus nearly triangular, pale except the thick black seam, with weak digitations around outer margin, with black blade-like marginal bristles on digitations. The tip is elongated with the apical bristle on a papilla. A strong black bristle present on the inner margin of the cercus near the tip. Hypandrium with one large brown hook-like tooth at tip and some tiny denticles on the dorsal margin (Figure 18C). The large brown tooth is resting on the brownish tip of a large pale membranous projections on both sides of the ventral margin of the epandrium (Figure 18B). Phallus bifurcate with a dorsal rounded swelling on the dorsal fork (Figure 18A). Tip of the ventral fork somewhat truncate (Figure 18E).

**Female**. Unknown.

###### Comments.

Loew (1864) gave a very detailed description of the male and although he mentioned the small swelling of the costa before the R_1_ reaches the costa, he did not indicate it on his drawings of the wing (Loew 1864: figure 3 and 3C). That caused more confusion. Having this characteristic *L.polychroma* resembles our *L.nodulata* that has however a larger swelling of the costa on the point where R_1_ joins the costa (Figs 16, 17) and it lacks the large brown tooth a the tip of the hypandrium. *L.zhangae* sp. n. from Bali has no broadening before the R_1_ joins the costa but identical armed hypandrium. *Lichtwardtiahirsutiseta* has a broad swelling much more in advance of the point where R_1_ meets the costa; its antenna is also much darker while entirely yellow in *L.polychroma*. Here again we did not dissect the specimen waiting for appropriate techniques to study the ancient DNA. Nevertheless we think that *L.polychroma* is conspecific with specimens from Cambodia that we describe above in more detail.

###### Distribution.

Cambodia, Sri Lanka.

##### Lichtwardtia zhangae

Taxon classificationAnimaliaDipteraDolichopodidae

Tang & Grootaert sp. n.

http://zoobank.org/7C6ADA9C-E5C2-457A-900D-74822F90DFBD

Lichtwardtia ziczac (Wiedemann, 1824) sensu Zhang, Masunaga & Yang, 2009: 199, figs 11–14. Re-description.

###### Etymology.

This species is dedicated to Dr. Lili Zhang of the IOZ Museum in Beijing who re-described and illustrated *Lichtwardtiaziczac* (Wiedemann) for the first time with detailed drawings.

###### Diagnosis.

Costa without swelling. Hypandrium with a large brown dorsal preapical tooth. Phallus smooth.

###### Description.

For a full description we refer to Zhang et al. 2009.

###### Comments.

In having the cross veins clouded, *Lichtwardtiaziczac* (Wiedemann) is distinctly different from *L.ziczac* sensu Zhang et al. 2009 and therefore we give a new name *Lichtwardtiazhangae* sp. n. to the species that she (re-) described from Bali. It is very closely related to *L.polychroma* from Sri Lanka and differs in the lacking of a swelling of the costa.

###### Distribution.

Bali (Indonesia).

#### Unplaced species

##### Lichtwardtia formosana

Taxon classificationAnimaliaDipteraDolichopodidae

Enderlein, 1912

Figs 19 , 20

Lichtwardtia formosana Enderlein, 1912: 407. Type locality: Taiwan (China).
Lichtwardtia taiwanensis Zhang, Masunaga & Yang, 2009: 199, figs 6–10. Type locality: Taiwan (China).
Lichtwardtia formosana Enderlein, 1912, in Selivanova, Negrobov & Yang, 2010: 144, re-description, figs 1–5.

###### Material examined.

CAMBODIA (all coll. RBINS): 10 males, Siem Reap prov., Angkhor, Preah Khan Temple, 24 January–21 February 2005, malaise trap in secondary forest (leg. Oul Yothin). 1 male; same provenance, 12 November–7 December 2005. 4 males, same provenance, 28 March–4 April 2006. 2 males, same provenance, 4–11 April 2006. 1 male; same provenance, 12 May 2006. 1 female, Siem Reap prov., garden Sam Vesna Centre, 6 June 2005, malaise trap (leg. Oul Yothin).

SINGAPORE (all coll. LKCNHM): 1 male, Semakau, 3 April 2012, sweeping along path in back mangrove (leg. P Grootaert). 1 male, Semakau, 12 July 2012, sweeping along path in mangrove (leg. Jayanthi Puniamoorthy & P Grootaert). 1 male, 2 females, West Coast Park, 7 December 2003; sweeping nets along shrubs along sandy beach (leg. P Grootaert). 1 male, 5 female, Sungei Buloh, 1–6 June 2005, malaise trap in mangrove (leg. P Grootaert). 5 female, same provenance the previous, 20–27 July 2005. 1 male, 2 females, Clementi Woods, sweeping nets along drains in park (leg. P Grootaert). 1 male, 1 female, Labrador Park, 3 April 2005, sweeping along drains (leg. P Grootaert).

###### Diagnosis.

Antenna entirely dark yellow. Mid coxa entirely darkened, hind coxa anteriorly with a rectangular brown spot. No thickening of the costa present. Wing nearly hyaline, lightly tinged brown. Apex of phallus looking smooth though microscopic small denticles are present.

###### Description.

**Male**. *Body* length 3.8 mm, wing 3.5 × 1.2 mm.

*Head* dark metallic green, with thick pale pollinosity; face slightly raised, frons and face both with thick pale pollinosity, gradually narrowed downward. Hairs and bristles on head black except postocular bristles pale. Antenna dark yellow; postpedicel nearly triangular, blunt apically, 1.4 times as long as wide, covered by short brown pubescence; arista-like stylus dorsal, black to dark yellow onwards, inserted at basal half of postpedicel, nearly as long as head width, black, feather-like, with long black pubescence, basal segment 0.5 times as long as apical portion of arista-like stylus. Proboscis brown, with black hairs; palpus yellow, with one short black apical bristle.

*Thorax* dark metallic green, with pale grey pollinosity. Hairs and bristles on thorax black; five strong dc, ten pairs of strong acr. Scutellum with two pairs sc, apical pair long strong, basal pair short and weak. Legs mainly yellows, except basal 2/3 of mid coxa brown, hind coxa with one brown spot, tip of hind tibia brown, tarsi yellow to brown onwards. Fore coxa anteriorly with five strong bristles at apical half, mid coxa anteriorly with four strong bristles, hind coxa with two short outer bristles. Fore and hind trochanters with one weak outer bristle at middle. Mid trochanter with two short weak outer bristles. Fore femur without distinct bristle. Mid and hind femora each with one preapical pv. Fore tibia with two short ad, two pd, one av, and three ap. Mid tibia with two ad, three pd and four ap. Hind tibia with six ad, four pd, two pv, and three ap. Hind tarsomere I with one short strong ad at apical third, one av at basal fifth and one short apical bristle. Relative lengths of tibia and five tarsomeres of legs LI : 2.5 : 1.1 : 0.5 : 0.4 : 0.3 : 0.3; LII : 3.0 : 2.0 : 1.0 : 0.8 : 0.5 : 0.3; LIII : 4.0 : 1.5 : 1.5 : 1.0 : 0.8 : 0.5. Wing nearly hyaline, lightly tinged brown; veins brown. M with fading M_2_, M_1_ with one short subvein. Crossvein dm-cu almost straight. CuAx ratio 1.0. Lower calypter pale with black hairs. Haltere pale.

*Abdomen* metallic green, with pale pollinosity. Hairs and bristles on abdomen black.

*Male terminalia*: Epandrium 2.0 times longer than wide (Figure 20); epandrial lobe three pale ap. Ventral surstylus with five long ap, dorsal surstylus with three ap and two digitations each with one apical bristle, of which one thin spinous, one rod-like; all pale except rod-like bristle brown. Cercus nearly triangular, pale except for the black marginal seam, with weak digitations around outer margin and with thin pale marginal bristle on digitations. Hypandrium simple. Phallus with a patch of minute spinules near apex (these spinules are visible like a darkened patch and can only be distinguished well under a light microscope).

**Female**. Has the same characteristics as the male: large rectangular black spot on the hind coxa and the tip of the hind tibia brown.

###### Comments.

*Lichtwardtiaformosana* is the only Oriental species known at the moment with a rectangular black (dark brown) patch on the hind coxa. In all other species the hind coxa is entirely yellow. *Lichtwardtiaformosana* looks different but has a double row of microscopic denticles ventrally on the tip of the phallus. Otherwise the shape of the postgonite is identical to the *L.nodulata* group. The cercus lacks a strong dorsal bristle at the inside.

###### Distribution.

China (Taiwan), Cambodia, Singapore.

**Figure 19.**
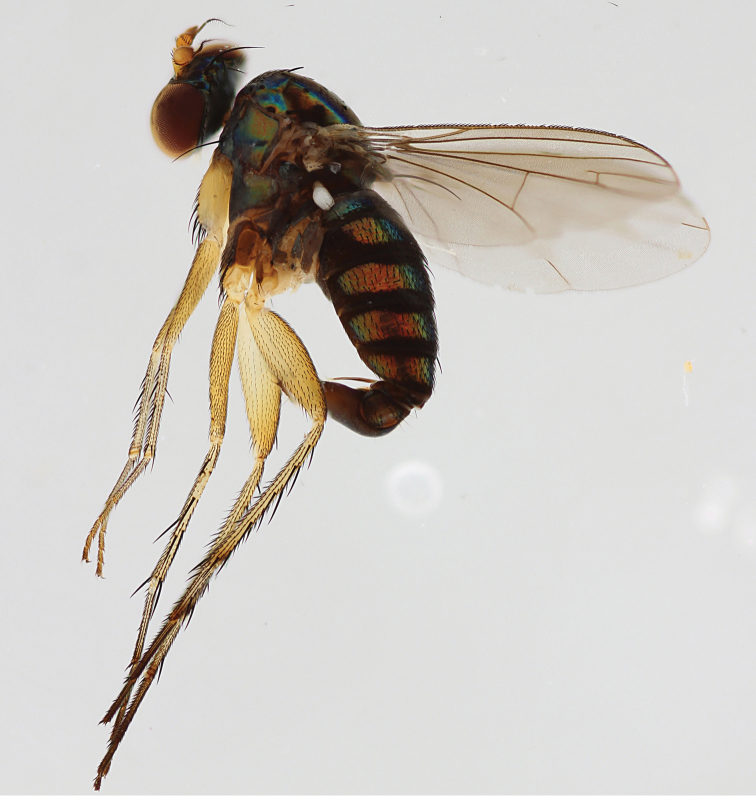
*Lichtwardtiaformosana* Enderlein male habitus (Singapore)

**Figure 20.**
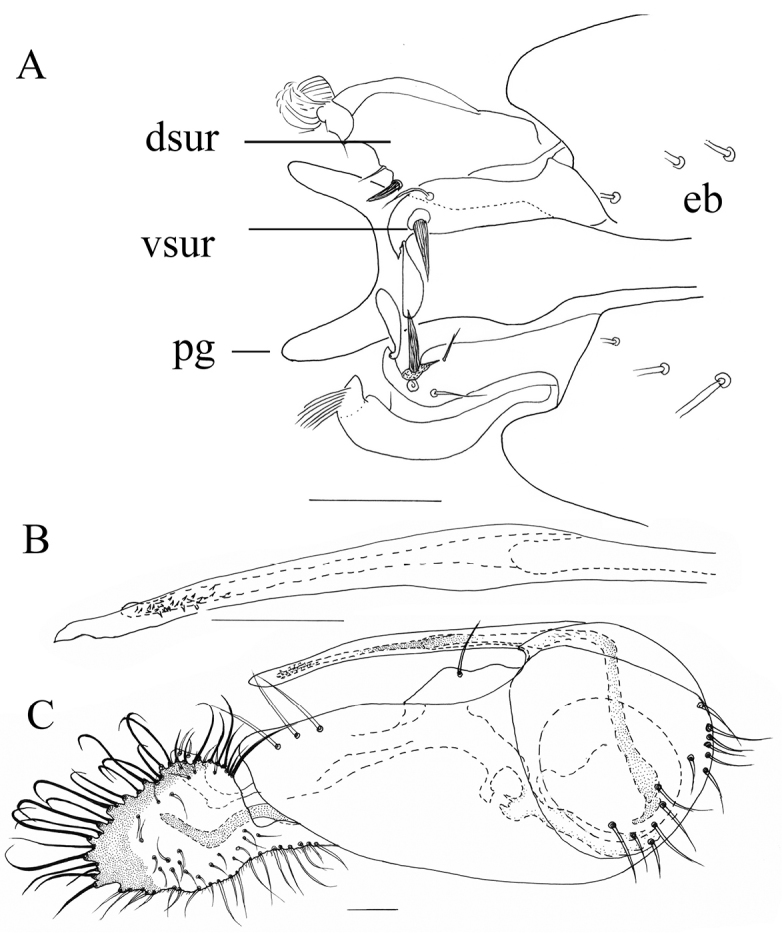
*Lichtwardtiaformosana* Enderlein male terminalia (Singapore): **A** ventral view on surstyli and cerci **B** tip phallus bearing microscopic denticles **C** lateral view epandrium. Scale bars: 0.1 mm.

##### Lichtwardtia hirsutiseta

Taxon classificationAnimaliaDipteraDolichopodidae

(de Meijere, 1916)

Figure 21

Rhagoneurus hirsutisetus de Meijere, 1916: 229. Male. Type locality Batavia (= Jakarta, Indonesia).

###### Material examined.

**Figure 21.**
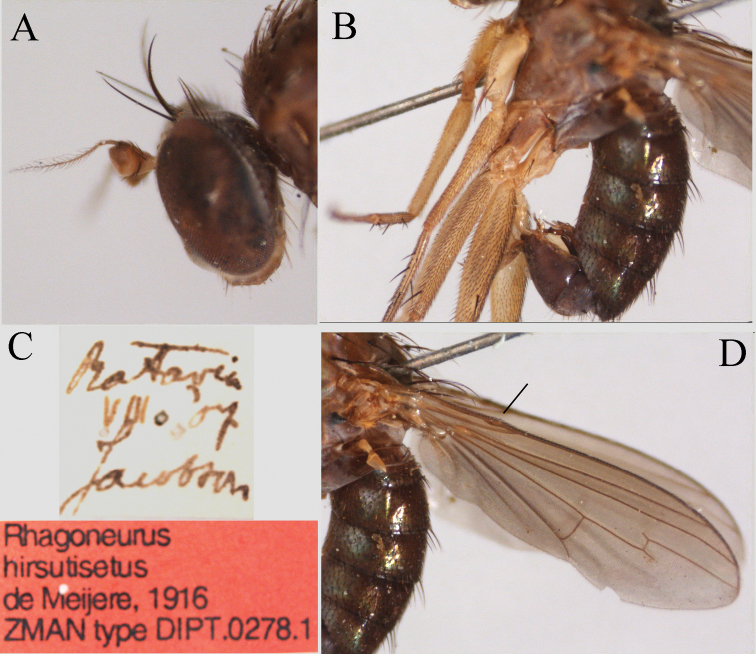
*Lichtwardtiahirsutiseta* (de Meijere, 1916) holotype male (Jakarta, Indonesia) (photograph by Mr Ben Brugge): **A** head lateral **B** thorax and abdomen lateral **C** labels **D** wing. Arrow indicates the swelling before R_1_ reaches the costa.

Holotype male Batavia, August, 1907, leg. Jacobson (Figure 21) Naturalis (Leiden, Netherlands). The male was not physically examined by us, only the photographs (courtesy of Ben Brugge).

###### Diagnosis.

A larger species (body length 4.5 mm; wing length 4.0 mm). Postpedicel mainly dark yellow, but blackish on dorsum and tip. Wing brown with cross veins not brownish seamed and costa with a distinct swelling well before R_1_ reaches the costa (Figure 21D). Hind coxa yellow (Figure 21B). Male terminalia with complicated twisted hypandrium and phallus (Figure 21B).

###### Comments.

As marked in the diagnosis *L.hirsutiseta* is quite unique in having a largely darkened postpedicel while other *Lichtwardtia* have generally an entirely yellow postpedicel. The swelling of the costa is also unique. It is well separated and before R_1_ reaches the costa. The complex male terminalia resemble *L.monstruosa* sp. n. but not *L.polychroma*.

##### Lichtwardtia infuscata

Taxon classificationAnimaliaDipteraDolichopodidae

Tang & Grootaert sp. n.

http://zoobank.org/D34616A1-6260-4145-894B-5EDEA83DABFA

Figs 22 , 23 , 24

###### Material examined.

**Holotype male** (coll. RBINS): CAMBODIA: Siem Reap prov., Angkhor, Preah Kahn Temple, 24 January–21 February 2006, Malaise trap in secondary forest (leg. Yothin Oul). **Paratypes**: 2 males, same provenance as holotype, 28 March–5 April 2005.

###### Diagnosis.

Wing anteriorly brown, grey below Cu and M_1_. Costa lacking a swelling. Postpedicel about as long as wide with rounded tip, apex brownish yellow, ventrally yellowish. Mid coxa anteriorly with a black stripe, posteriorly brown. Hind coxa entirely yellow. Phallus with strong black spines, phallus and hypandrium resting on a ventral protuberance of the epandrium. Cercus seamed with long, fine black and pale bristles.

###### Description.

**Male**. *Body* length 4.1 mm, wing 3.2 × 1.1 mm. Head dark metallic green, with thick pale pollinosity; face slightly raised, frons and face both with thick pale pollinosity, gradually narrowed downward. Hairs and bristles on head black but lower postocular bristles yellowish. Antenna yellow except but apical half of postpedicel brown; postpedicel nearly triangular, 1.2 times as long as wide; arista-like stylus black, almost as long as head width, feather-like, with long regular pubescence. Proboscis dark yellow, with black hairs; palpus very small, dark yellow, with one short black apical bristle.

**Figure 22.**
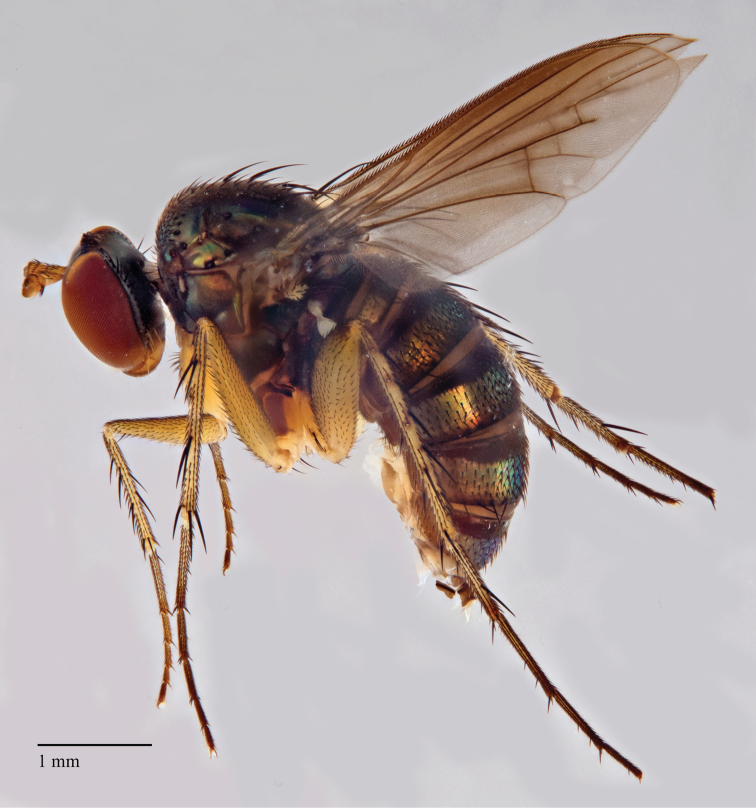
*Lichtwardtiainfuscata* Tang & Grootaert, sp. n. Holotype male (terminalia removed). Cambodia (photograph by Ms Maimon Hussin).

*Thorax* dark green, with pale grey pollinosity. Hairs and bristles on thorax black; five strong dc, ten pairs of strong acr. Scutellum with two pairs sc, apical pair long strong, basal pair short and weak. Legs mainly yellow. Mid coxa anteriorly with a black stripe, posteriorly brown. Hind coxa entirely yellow. Fore tarsomere I brown, apical fifth of each hind tarsomere brownish. Fore and mid coxae anteriorly with rows of bristle-like hairs, and four strong ap, hind coxa with two outer bristles, basal one strong, apical one relatively weak. Mid and hind trochanters both with four short weak ap. Fore femur with one weak preapical av. Mid femur with one strong pd and one preapical av. Hind femur with one strong pd at apical quarter. Fore tibia with two ad, three pd, one av and three ap. Mid tibia with two ad, four pd and four ap; all long strong. Hind tibia with four ad, four pd (of which one preapical), one d, two pv and three ap. Fore tarsomere I with one short av at base. Hind tarsomere I with one strong ad at apical third, one short ad at base and two short apical bristle. Relative lengths of tibia and five tarsomeres of legs LI : 7.5 : 4.5 : 1.8 : 1.3 : 1.0 : 1.0; LII : 11.2 : 6.3 : 3.0 : 2.5 : 1.5 : 1.3; LIII : 12.5 : 5.0 : 5.2 : 3.8 : 2.5 : 1.5. Wing anteriorly brownish infuscate (Figure 22A) including the cross veins; veins brown. M with fading M_2_, M_1_ with one short subvein. Crossvein dm-cu straight. CuAx ratio 1.0. Lower calypter pale with black hairs. Haltere pale.

*Abdomen* metallic green, with pale pollinosity. Hairs and bristles on abdomen black.

*Male terminalia*: Epandrium 2.0 times longer than wide; epandrial lobe with three long pale bristles. Ventral surstylus with five ap, of which ventral most apical bristle rod-like and others spinous; dorsal surstylus both with five short nail-like ap and one long digitation close to dorsal margin which with one long spinous bristle. Phallus with strong black spines, phallus, and hypandrium resting on a ventral protuberance of the epandrium. Cercus nearly triangular, pale except the thick black ring, with weak digitations around outer margin, with long pale marginal bristles on digitations. Hypandrium simple. Phallus strong with strong black denticles (Figure 24A).

**Female**. Unknown.

###### Etymology.

From Latin infuscāre to darken.

###### Comments.

It is not clear if *L.infuscata* sp. n. belongs to the *L.dentalis* –group though it has the tip of the phallus ventrally denticulate and a simple hypandrium. The cercus is more elongate and lacks a dorsal bristle at the inside. Others see comments under *L.ziczac*.

###### Distribution.

Cambodia.

**Figure 23.**
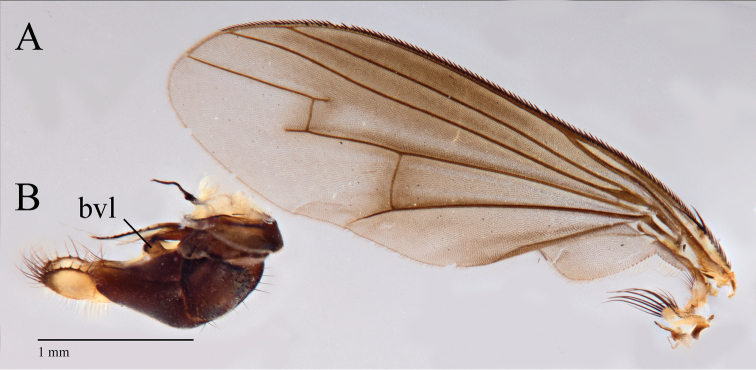
*Lichtwardtiainfuscata* Tang & Grootaert, sp. n. Paratype male. **A** Wing **B** Male terminalia (photograph by Ms Maimon Hussin).

**Figure 24.**
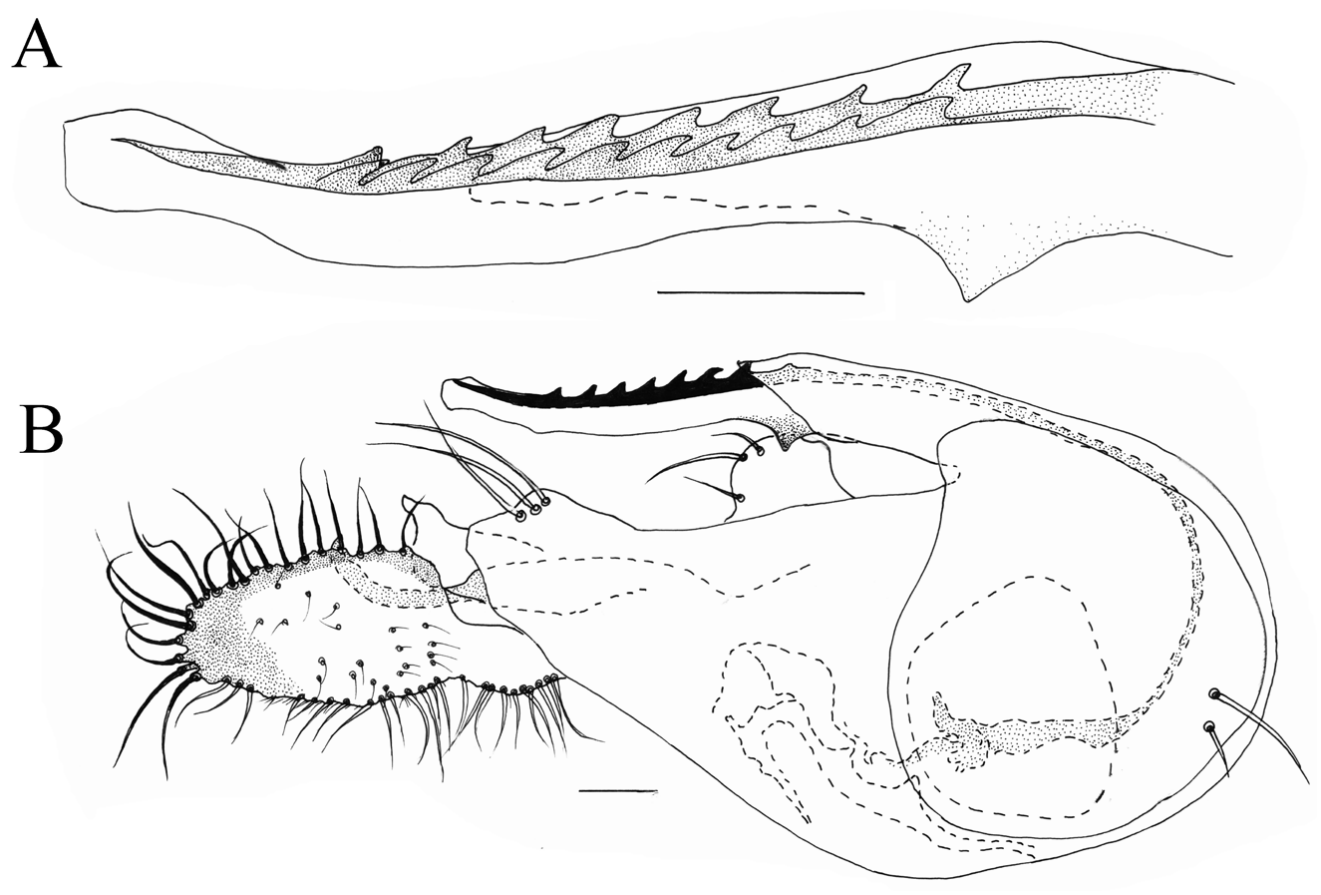
*Lichtwardtiainfuscata* Tang & Grootaert, sp. n. Male terminalia: **A** Tip phallus **B** epandrium lateral. Scale bars: 0.1 mm.

#### The nomina dubia

##### Lichtwardtia coxalis

Taxon classificationAnimaliaDipteraDolichopodidae

Kertész, 1901

Rhagoneurus coxalis Kertész, 1901: 411. Female. Type locality: Singapore.

###### Material.

The type was probably conserved at the Hungarian Museum for Natural History. It is no longer in their collections and probably destroyed (Foldvary and Papp 2007).

###### Diagnosis from Kertész.

Hind coxa largely blackish brown. Hind femur without preapical bristle and hind basal tarsomere lacking long bristles. Cross veins not brownish seamed.

###### Comments.

It is likely that this species is one of the four species that we actually recorded in Singapore. It is not *L.singaporensis* sp. n. since it has the cross veins brownish seamed and the hind coxa yellowish. *L.nodulata* sp. n. has a broad swelling where R_1_ joins the costa and the hind coxa is yellowish (Figure 6). *L.semakau* sp. n. has also a yellow hind coxa (Figure 8). *L.formosana* Enderlein is the most plausible candidate because it is particular in having only the anterior half of the hind coxa dark brown (rectangular sclerotisation) while the posterior part is yellowish to yellowish brown (Figure 19). Maybe Kertész meant this in stating that *L.coxalis* has “auch die hinterhüften in grosser ausdehnung schwarzbraun”. However the shape of the black sclerotisation is so remarkable. In his key, Kertész (1901) described the similar shape of the mid coxa of *L.polychroma* but not for the hind coxa. *L.formosana* from Singapore possesses an anterior preapical bristle on the hind femur (lost on Figure 19) that is according to Kertész not present in *L.coxalis*.

Since it was described on the basis of a female and that the holotype seems to be lost, we think it is not appropriate to sink *L.formosana* as a junior synonym having its holotype conserved. The confusion is bigger since de Meijere (1912) quotes *L.coxalis* (as *Rhagoneurus*) from “Neu Guinea” but this is a misreading because Kertész clearly says that he obtained a female from Singapore by the courtesy of his friend Biro. Meanwhile, the description by Kertész is too simple to acquire any further comparison. Therefore, at this moment, *L.coxalis* is considered as a nomen dubium.

##### Lichtwardtia ziczac

Taxon classificationAnimaliaDipteraDolichopodidae

(Wiedemann, 1824)

Figure 25

Dolichopus ziczac Wiedemann, 1824: 40. Female. Type locality: India Orientalis
Dolichopus ziczac Wiedemann, 1830: 232
Dolichopus zickzack Wiedemann, 1824. Male in Becker 1922: 8, figure 1. Non ziczac sensu Wiedemann 1824
Lichtwardtia ziczac (Wiedemann, 1824) sensu Zhang, Masunaga & Yang, 2009: 199, figs 11–14.

###### Material examined.

Holotype female, India Orientalis, on pin in collection ZMUC (Copenhagen).

###### Diagnosis.

**Figure 25.**
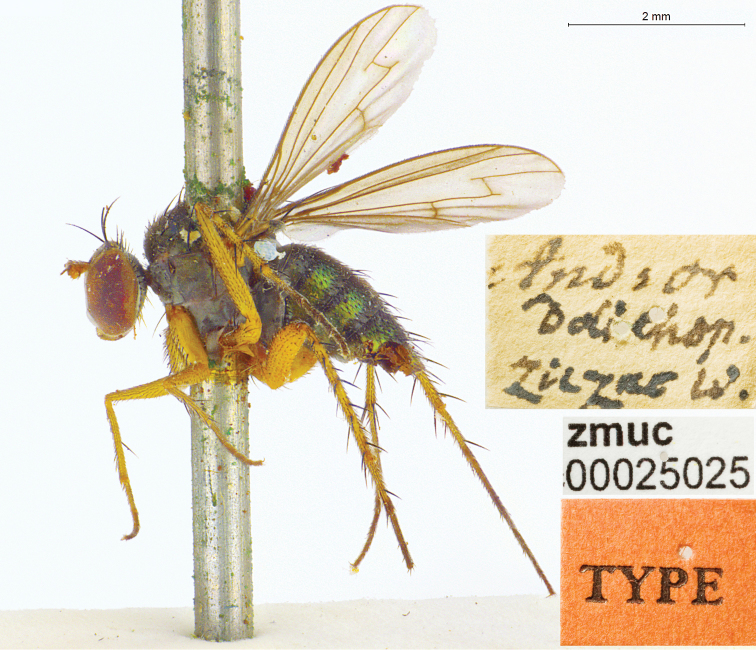
*Lichtwardtiaziczac* (Wiedemann, 1824) holotype female (photograph by Dr Thomas Pape).

Female. A medium-sized species (body: 4 mm; wing: 3.2 mm). Wing hyaline with anterior border faintly brownish and cross veins brownish seamed. No swelling of the costa before or at the point where R_1_ joins the costa. The ratio of the proximal section of M_1_, and the distal section is 0.4/0.6 (Figure 25). Thus the distal section is much longer than the proximal section. Fore coxa yellow, mid coxa brown and hind coxa yellowish.

###### Comments.

*Dolichopusziczac* was described by Wiedemann (1824) on the basis of a female collected in India Orientalis. No precision is given about the locality so that the type locality could be everywhere in the Oriental region ranging from Pakistan to New Guinea at that time. The description of the species in the work of Wiedemann (1824) is very short, but fortunately the holotype female is still well preserved in the collections of the Zoological Museum in Copenhagen.

Becker (1922) knew about this specimen but did not see it. A colleague described it to him in a letter and Becker was sure that the specimens that he had in his own collection or had seen at the Hungarian Museum from Taiwan (China), India, Bangladesh, Rangoon (Myanmar), Ceylon (Sri Lanka), and the Bismarck Archipelago (Papua New Guinea) were all the same species. The range is thus also very wide according to Becker. He gave a description of a male but did not mention the origin of the male. Hence we cannot rely on his re-description that fits to quite a number of species. At the same time he put *Rhagoneuruscoxalis* Kertész, 1901, *Lichtwardtiaformosana* Enderlein, 1912 and *Rhagoneuruspolychromus* Loew, 1864 all *Lichtwardtia* species as junior synonyms of *L.ziczac*. Now we see that all are good species and we re-establish here their names as valid species. The previous holotype female is examined (Figure 25). It is not clear why Becker (1922) changed the name *ziczac* to *zickzack*.

Brownish seams along the cross veins are not very common in *Lichtwardtia* and actually only known in *L.ziczac* and *L.singaporensis* sp. n. The ratio of the proximal section of M_1_, and the distal section is however 0.435/0.564. Thus the distal section is not as long as in *L.ziczac*. We do not consider both species as conspecific for the moment because in the near future it might be possible to extract ancient DNA from the holotype without using destructive techniques. This can be decisive about the status of both species. We propose to consider *L.ziczac* (Wiedemann) from *terra incognita* as a *nomen dubium* and not to complicate again the taxonomy of *Lichtwardtia* by applying the name *ziczac* to the male of *singaporensis* without genetic information. Remarkable is that among the more than 200 *Lichtwardtia* specimens belonging to six species that we found in Seam Reap, no specimens with brownish seamed cross veins were found.

#### Key to males of Oriental *Lichtwardtia*

**Table:** 

1	Hind coxa with anteriorly half or basal half brownish	**2**
–	Hind coxa entirely yellow	**3**
2	Anterior half of hind coxa with a dark brown rectangular area that bears the two black exterior bristles; cross veins not seamed brownish. Apex of phallus looking smooth though microscopic small denticles are present (Figure 20) [Taiwan (China), Cambodia, Singapore]	***formosana* Enderlein**
–	Hind coxa with basal half brownish and cross veins brownish seamed. Phallus ventrally with strong denticles, hypandrium unarmed. (Figs 10–11) (Singapore)	***singaporensis* sp. n.**
3	Wing with a colour pattern (Figure 23). Phallus with strong black spines, phallus, and hypandrium resting on a ventral protuberance of the epandrium (Figure 24). (Cambodia)	***infuscata* sp. n.**
–	Wing uniformly hyaline, greyish or brownish tinged but without pattern. Male terminalia various	**4**
4	Costa with a swelling before or at the point where R_1_ joins the costa	**5**
–	Costa without swelling	**10**
5	A distinct brown swelling of the costa where R_1_ joins the costa (Figure 6). Phallus with two rows of black denticles on ventral half, anterior row only with four small sparse denticles, posterior row with eight dense denticles (Figure 7). (Singapore)	***nodulata* sp. n.**
–	Costa widened before R_1_ reached costa (Figure 21 D)	**6**
6	Costa widened distinctly before R_1_ joins costa (Figure 21D); postpedicel black except for yellowish base. Male terminalia with complicated twisted hypandrium and phallus (Figure 21 B) (Java, Indonesia)	***hirsutiseta* de Meijere**
–	Costa widened just before tip of R_1_ (Figure 4); postpedicel yellowish, sometimes dorsally darker	**7**
7	Tip of phallus with black ventral denticles (Figure 4). Tip of hypandrium without a preapical tooth or not bifurcate (Yunnan China, Thailand, Cambodia, Singapore)	**8**
–	Tip of phallus lacking ventral denticles; tip of hypandrium with a large brown tooth (Figure 18) or tip hypandrium bifurcate (Figure 13)	**9**
8	Marginal bristles on cercus strong, black (Figure 5). A distinct swelling on costa at level of R_1_. Tip of phallus ventrally with a number of denticles. Hypandrium unarmed (Figs 4, 5)	***dentalis* Zhang et al.**
–	Marginal bristles on cercus weak, pale. Swelling of costa weak. Hypandrium simple and smooth, with no denticle; phallus with double rows of spinules on ventral half (Figure 3)	***cambodiensis* sp. n.**
9	Tip of hypandrium with a single large brown preapical tooth (Figs 18, 19) (Sri Lanka, Cambodia, China)	***polychroma* (Loew)**
–	Tip of hypandrium dorsally curved, bifurcate; with a long black appendage near middle of hypandrium (Cambodia)	***conspicabilis* sp. n.**
10	Hypandrium simple (Figure 9)	***semakau* sp. n.**
–	Hypandrium with tooth-like extensions	**11**
11	Hypandrium with a large preapical brown tooth (Bali)	***zhangae* sp. n.**
–	Hypandrium with ventral and dorsal black saw-toothed extensions (Figure 15) (Cambodia)	***monstruosa* sp. n.**

## General discussion

### Diversity

With twelve species known now in the Oriental Realm, the genus *Lichtwardtia* is apparently quite species rich especially in view of the poor local sampling. Here we added eight species new for science. Interestingly, the external non-genitalic morphology is not very diverse but the male terminalia are distinctly different. It is perhaps too early to place all species into species groups but some closely related species can be distinguished.

A first large species group is the *L.nodulata* group with *cambodiensis* sp. n., *L.dentalis* Zhang et al., *L.nodulata* sp. n., *L.semakau* sp. n., and *L.singaporensis* sp. n.

The sister-group *L.polychroma* and *L.zhangae* are probably related to the *nodulata*-group in having a similarly shaped cercus with some strong flattened marginals and a dorsal marginal that shifted to the inside. However the postgonite is tubiform while it is broad with bifid tip in the *L.nodulata* group. Moreover the tip of the phallus is not ventrally denticulate and the hypandrium has a strong subapical dorsal hook. Provisionally we put both species in the *polychroma*-group.

*Lichtwardtiaconspicabilis* sp. n. and *L.monstruosa* sp. n. seem to be related in having a forked hypandrium and a forked phallus, thus lacking the ventral denticles on the tip of the phallus in the *nodulata* group. Provisionally we place both species in the *conspicabilis*-group.

**Figure 26.**
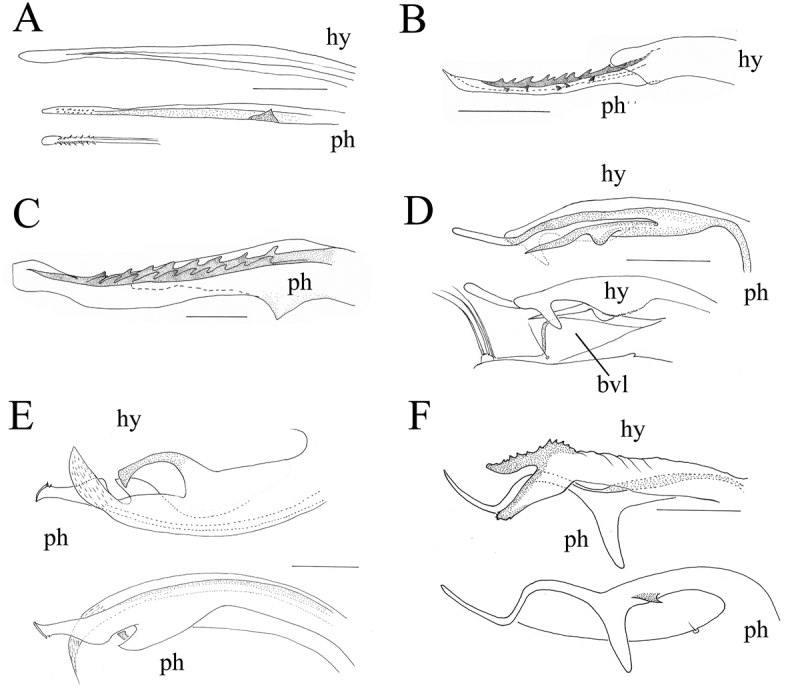
Variation in morphology of hypandrium and phallus in Oriental *Lichtwardtia* species. Scale bars: 0.1 mm. **A***formosana*-group: *L.formosana*: phallus slender, hidden in hypandrium. Tip of phallus ventrally with a double row of microscopic denticles **B***nodulata*-group: *L.nodulata*: tip of phallus ventrally with strong denticles. Tip of phallus never retracted in the hypandrium **C***nodulata*-group: *L.infuscata*: dorsal spine present at base of exposed part of phallus **D***polychroma*-group: *L.polychroma* hypandrium with a large dorsal preapical tooth resting on a large basoventral epandrial lobe. Phallus robust, forked **E***conspicabilis*-group: *L.conspicabilis*: tip of hypandrium membranous and with a right extension. Phallus robust with a forked tip **F***conspicabilis*-group: *L.monstruosa* tip of forked hypandrium with denticles. Tip of phallus slender but with a large dorsal tooth near middle of phallus.

It is not clear if *L.infuscata* sp. n. belongs to the *L.nodulata* group though it has the tip of the phallus ventrally denticulate and a simple hypandrium. The cercus is more elongate and lacks a dorsal bristle at the inside. *L.formosana* looks different but has a double row of microscopic denticles ventrally on the tip of the phallus (Figure 26A). Otherwise the shape of the postgonite is identical to the *L.nodulata*-group. The cercus lacks a strong dorsal bristle at the inside. *L.hirsutiseta* is quite unique as its callus of the costa is well separated and before R_1_ reaching the costa. The complex male terminalia resemble *L.monstruosa* sp. n., but not *L.polychroma*. Thus we leave it unplaced.

**Figure 27.**
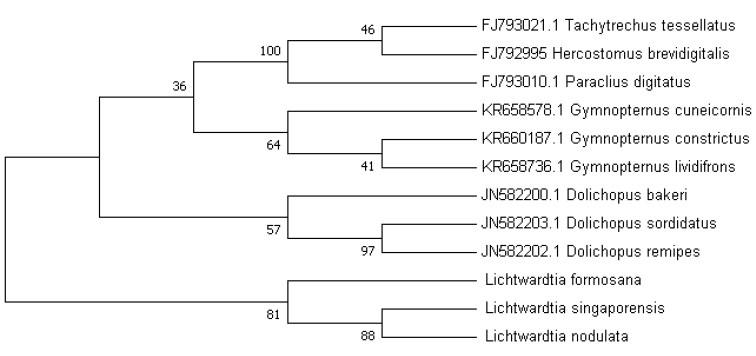
Phylogeny of Singaporean *Lichtwardtia* species and some other species of Dolichopodinae.

At the moment we could only provide barcodes of the species from Singapore (uploaded to GenBank, with accession number MH536852-MH536856). The material from Cambodia was collected in ethanol of poor quality and therefore not suitable for sequencing. The optimal tree with the sum of branch length = 0.27350170 is shown. The percentage of replicate trees in which the associated taxa clustered together in the bootstrap test (1000 replicates) are shown next to the branches. The tree is drawn to scale, with branch lengths in the same units as those of the evolutionary distances used to infer the phylogenetic tree. As can be seen in Figure 27 the species *L.nodulata* sp. n. and *L.singaporensis* sp. n. cluster well while *L.formosana* is more distant, as what it inferred from morphological study. The species with simple phallus (*L.formosana*) is shown to have a more primitive placement, which clearly demonstrates that the species with more complex male genitals are the derived species, the characters are tending to be more complicated by the evolution. The phylogeny also support the monophyly of *Lichtwardtia*.

### From bare to heavily armed male terminalia

As can be seen on Figure 26 it seems as if a various diversification in armament of the phallus and hypandrium took place in Oriental *Lichtwardtia* species. There have been such finding in beetles and bugs, but never had such a phenomenon reported from Diptera (Crudgington and Siva-Jothy 2000; Matsumura et al. 2017; Rōnn et al. 2007; Stutt and Siva-Jothy 2001). It has been proved that males with longer genital spines were more successful in gaining fertilisations, providing experimental evidence that male genital morphology influences success in post-copulatory reproductive competition (Hotzy et al. 2012). Normally, three potential benefits are speculated for such specialisation: last-male sperm precedence, suboptimal re-mating frequencies for the maintenance of female fertility, and reduced longevity and reproductive success in females (Stutt and Siva-Jothy 2001). These are all plausible explanations in our case. On the other hand no distinct male secondary sexual characters so typical for dolichopodids are present such as coloured whiskers, flag-like tarsi usually used in display to the females.

## Supplementary Material

XML Treatment for Lichtwardtia

XML Treatment for Lichtwardtia nodulata

XML Treatment for Lichtwardtia cambodiensis

XML Treatment for Lichtwardtia dentalis

XML Treatment for Lichtwardtia nodulata

XML Treatment for Lichtwardtia semakau

XML Treatment for Lichtwardtia singaporensis

XML Treatment for Lichtwardtia conspicabilis

XML Treatment for Lichtwardtia conspicabilis

XML Treatment for Lichtwardtia monstruosa

XML Treatment for Lichtwardtia polychroma

XML Treatment for Lichtwardtia polychroma

XML Treatment for Lichtwardtia zhangae

XML Treatment for Lichtwardtia formosana

XML Treatment for Lichtwardtia hirsutiseta

XML Treatment for Lichtwardtia infuscata

XML Treatment for Lichtwardtia coxalis

XML Treatment for Lichtwardtia ziczac
